# Reframing How Physical Activity Reduces The Incidence of Clinically-Diagnosed Cancers: Appraising Exercise-Induced Immuno-Modulation As An Integral Mechanism

**DOI:** 10.3389/fonc.2022.788113

**Published:** 2022-03-14

**Authors:** Annabelle Emery, Sally Moore, James E. Turner, John P. Campbell

**Affiliations:** ^1^ Department for Health, University of Bath, Bath, United Kingdom; ^2^ Department of Haematology, Royal United Hospitals Bath NHS Foundation Trust, Bath, United Kingdom

**Keywords:** cancer, exercise, physical activity, exercise oncology, exercise immunology

## Abstract

Undertaking a high volume of physical activity is associated with reduced risk of a broad range of clinically diagnosed cancers. These findings, which imply that physical activity induces physiological changes that avert or suppress neoplastic activity, are supported by preclinical intervention studies in rodents demonstrating that structured regular exercise commonly represses tumour growth. In Part 1 of this review, we summarise epidemiology and preclinical evidence linking physical activity or regular structured exercise with reduced cancer risk or tumour growth. Despite abundant evidence that physical activity commonly exerts anti-cancer effects, the mechanism(s)-of-action responsible for these beneficial outcomes is undefined and remains subject to ongoing speculation. In Part 2, we outline why altered immune regulation from physical activity - specifically to T cells - is likely an integral mechanism. We do this by first explaining how physical activity appears to modulate the cancer immunoediting process. In doing so, we highlight that augmented elimination of immunogenic cancer cells predominantly leads to the containment of cancers in a ‘precancerous’ or ‘covert’ equilibrium state, thus reducing the incidence of clinically diagnosed cancers among physically active individuals. In seeking to understand how physical activity might augment T cell function to avert cancer outgrowth, in Part 3 we appraise how physical activity affects the determinants of a successful T cell response against immunogenic cancer cells. Using the cancer immunogram as a basis for this evaluation, we assess the effects of physical activity on: (i) general T cell status in blood, (ii) T cell infiltration to tissues, (iii) presence of immune checkpoints associated with T cell exhaustion and anergy, (iv) presence of inflammatory inhibitors of T cells and (v) presence of metabolic inhibitors of T cells. The extent to which physical activity alters these determinants to reduce the risk of clinically diagnosed cancers – and whether physical activity changes these determinants in an interconnected or unrelated manner – is unresolved. Accordingly, we analyse how physical activity might alter each determinant, and we show how these changes may interconnect to explain how physical activity alters T cell regulation to prevent cancer outgrowth.

## Introduction

It is well established that regularly undertaking a large volume of physical activity is associated with a reduced risk of developing a wide range of clinically diagnosed cancers (1, 2). In addition to these common observations, a plethora of animal studies show that regular exercise commonly represses tumour growth (3, 4). In Part 1 of this review, key epidemiology and preclinical evidence linking physical activity or structured exercise training and cancer risk or tumour growth is summarised.

Despite abundant preclinical and human epidemiology evidence indicating that physical activity commonly exerts anti-cancer effects, the mechanism(s) responsible for these outcomes remains unknown and is the subject of ongoing discussion (5–7). In Part 2, we summarise why regulation of the immune system – and specifically T cells – is likely to play a critical role in mediating the reduced incidence of clinically diagnosed cancers among physically active individuals. We do this by summarising how physical activity appears to modulate the path of the cancer immunoediting process (8). In doing so, we highlight that the anti-cancer effects of physical activity may ultimately be derived from augmented immune effector responses against cancer cell clones which have mutated to become immunogenic. We posit that this predominantly leads to the containment of cancers in a ‘precancerous’ or ‘covert’ equilibrium state – thus delaying and/or averting a clinical cancer diagnosis.

Whilst it is increasingly apparent that physical activity may regulate T cell function to contain cancers, it remains unresolved how physical activity modifies the regulation of T cells to achieve this outcome. Accordingly, in Part 3 we evaluate how physical activity may alter determinants of a successful T cell response against tumour cells. The cancer immunogram – an established framework for predicting T cell success against immunogenic tumour cells (9) – specifies five T cell-intrinsic factors that dictate success, these are: (i) general T cell status in blood, (ii) T cell infiltration to tissues, (iii) presence of immune checkpoints associated with T cell exhaustion and anergy, (iv) presence of inflammatory inhibitors of T cells and, (v) presence of metabolic inhibitors of T cells. The extent to which physical activity alters these determinants to reduce the risk of clinically diagnosed cancers – and whether physical activity changes these determinants in an interconnected or unrelated manner – is unknown. Thus, in Part 3, we analyse each of these determinants in turn, and we then show how these determinants may individually, or in combination, explain how physical activity alters T cell regulation, resulting in more effective elimination of immunogenic cancer cells.

## Part 1: Regular Physical Activity Reduces the Risk of Clinically Diagnosed Cancers in Humans and Slows the Growth of Experimentally-Induced and Spontaneous Tumours in Rodents

Part 1 summarises findings from human epidemiology studies which consistently show that performing regular physical activity reduces the risk of an array of clinically diagnosed cancers. Additionally, Part 1 provides a synopsis of key findings from rodent studies which contribute to our understanding by enabling comparisons of cancer development in exercising rodents *vs.* non-exercising controls. Together, this evidence demonstrates that physical activity (in human epidemiology studies) and regular exercise (in rodent studies) commonly suppresses cancer growth.

### Physical Activity and Risk of Clinically Diagnosed Cancers: Key Human Epidemiology Evidence

A plethora of large epidemiology studies have investigated self-report physical activity and the risk of clinically diagnosed cancers. Historically, investigation of the relationship between physical activity level and clinical cancer risk was restricted to highly incident cancers, and these studies report reduced incidence of clinically-confirmed breast, colon, and prostate cancers in more physically active persons (10–12). More recently, meta-analyses of cohort studies capturing physical activity level and cancer incidence have facilitated the incorporation of less prevalent cancer types into statistical modelling. A landmark meta-analysis of 12 prospective cohort studies, including 1.44 million participants and >180,000 cancer cases, showed that the risk of 13 different cancers was lower in the 90^th^ *vs.* 10^th^ percentile of self-reported leisure-time physical activity (2). Negative associations between physical activity and risk of both solid and non-solid tumours across a range of tissue types and anatomical locations were identified, including oesophageal adenocarcinoma, myeloid leukaemia, multiple myeloma, and cancer of the liver, lung, kidney, colon, rectum, head and neck, bladder, breast, gastric cardia, and endometrium (2). Similarly, a meta-analysis including 770,000 cancer cases revealed that individuals undertaking the most physical activity had reduced risk of developing meningioma and cancer of the colon, breast, endometrium, lung, oesophagus, and pancreas, compared to individuals performing the least physical activity (13). Furthermore, a systematic review identified moderate-strong evidence for physical activity to reduce the risk of clinically diagnosed bladder, breast, colon, endometrial, oesophageal, gastric, renal, and lung cancers (14).

There also appears to be a dose-response relationship between physical activity and clinical cancer incidence. In a key study that furthered the analysis of physical activity-responsive cancers previously identified (2) – excluding lung cancer due to confounding effects of smoking (2) – an inverse linear dose-response relationship was found between weekly physical activity volume and risk of the most commonly diagnosed cancers of the breast, colon, endometrium, and head and neck, and oesophageal adenocarcinoma, with the greatest protection seen with >30 MET^1^-hours/week (1) – equivalent to 2-4x World Health Organisation (WHO) physical activity guidelines for 150-300 minutes of moderate or 75-150 minutes of vigorous intensity physical activity per week for adults, which is equivalent to 7.5-15 MET-hours/week (16). It is also notable that participation in more vigorous intensity physical activity appears to exert greater protection against clinical cancer incidence than lower intensities. For example, it was shown in a meta-analysis that vigorous intensity physical activity reduced clinically diagnosed breast cancer risk by 14%, compared to 3% for moderate intensity physical activity (17).

Epidemiology studies have also investigated the relative risk of cancer mortality according to physical activity intensity. Indeed, one study has shown – using data from 4672 participants who were cancer-free at physical activity assessment, of whom 326 died from cancer – that engaging in vigorous intensity physical activity reduced the risk of cancer mortality by 64% compared to those who performed no physical activity, where smaller risk reductions of 42% and 44% were seen for light and moderate intensity physical activity, respectively (18). Another study, including 2560 male participants and 181 cancer deaths, showed that the risk of cancer mortality was reduced by a further 20% when ≥30 minutes daily of physical activity was performed at a vigorous intensity (69% risk reduction) compared to moderate intensity (49% risk reduction) (19). Importantly, studies demonstrating the association between physical activity and cancer mortality indicate that the effects of physical activity are not finitely bound to reducing the risk of a cancer diagnosis, but instead, that the anti-cancer effects of physical activity can exist at all stages in the trajectory of cancer growth. Moreover, the risk of pan-cancer mortality decreases as physical activity level increases, with no upper limit observed (20). The maximum physical activity dose captured – >75 MET-hours/week, equivalent to 5-10x WHO physical activity guidelines – was associated with the greatest risk reduction (31%) in cancer mortality compared to the reference group reporting 0 MET-hours/week of physical activity (20).

Whilst this review highlights a potentially greater efficacy of very high doses of physical activity in reducing the risk of a clinical cancer diagnosis, it is important from a public health perspective to note that the risk of developing many cancers including breast, colon, endometrial, kidney, and liver cancer, multiple myeloma and non-Hodgkin lymphoma (1) and the risk of pan-cancer mortality (20) is still reduced in those achieving WHO physical activity guidelines of 7.5 MET-hours/week. Though, as greater protection is observed with higher amounts of physical activity, the American Cancer Society advocates 150-300 minutes of moderate intensity or 75-150 minutes of vigorous intensity physical activity per week to reduce the risk of a clinical cancer diagnosis (21). Importantly, under the recently updated WHO guidelines, resistance exercise is recommended for all age groups (16), however, an absence of evidence precluded recommendations for resistance exercise in American Cancer Society cancer prevention guidance (21). We highlight later in Part 3, that skeletal muscle may play a role in dictating the anti-cancer effects of physical activity.

On the balance of evidence, a dose-response relationship appears to exist between physical activity and a reduced risk of cancer incidence and mortality, and this relationship may be driven by the intensity and/or volume of physical activity. However, whether or not these beneficial effects are driven by direct physiological changes arising *during* individual bouts of exercise, or by an indirect effect arising as a biproduct of undertaking large volumes of exercise – such as changes to muscle mass, basal inflammation, or other factors – are not yet clear, and are discussed later in Parts 2 and 3.

#### Confounding Factors in Human Epidemiology Studies

It is important to acknowledge and discount the possibility that lower cancer incidence and mortality observed among people reporting high volumes of physical activity could be driven by a ‘clustering’ of favourable lifestyle factors. For example, physically active people may also consume a balanced diet, maintain a healthy body composition, and exhibit reduced exposure to lifestyle-associated carcinogens, such as smoking and alcohol intake. Whilst certain confounding factors (e.g., alcohol intake and dietary composition) are not often controlled for, recent epidemiology studies indicate that the reduced risk of clinically diagnosed cancers in physically active individuals appears to arise independently of smoking and obesity (1, 2).

Associations between physical activity and cancer risk withstand adjustment for smoking status for cancers of the liver, kidney, colon, rectum, head and neck, bladder, breast, gastric cardia, and endometrium, and for oesophageal adenocarcinoma and myeloid leukaemia (2). For other cancers like multiple myeloma, apparent detrimental effects of physical activity in current smokers, yet protective effects in former and never smokers, may have occurred due to chance in the presence of small case numbers (2). Further, while a null association between physical activity level and lung cancer risk was seen for never smokers (2), it is notable that only 10-15% of lung cancers are caused by factors other than smoking (22), and therefore the association with physical activity may have been lost due to low statistical power. Accordingly, for this reason, lung cancer was not included in a later follow-up study by the same group investigating dose-response relationships between physical activity and clinical cancer risk (1).

With regards to the assessment of obesity as a confounding variable, associations between physical activity and cancer risk are commonly adjusted for body mass index (BMI) in statistical modelling. Results from an aforementioned landmark meta-analysis indicated that the apparent benefits of physical activity withstood adjustment for BMI for all cancer types, except endometrial cancer (2). As up to 57% of endometrial cancer cases are attributable to a high BMI, indicative of overweight and obesity (23), it may be that low case numbers with BMI <25 kg/m^2^ in the aforementioned study explains the lost association between physical activity and lower risk of clinically diagnosed endometrial cancer. The previously described finding of BMI-independent effects of physical activity on reducing the risk of cancer incidence (2) has been replicated recently (1), reinforcing the possibility that the protective effects of physical activity to reduce clinical cancer risk are largely independent of obesity. Taken together, the effects of physical activity appear to act independently of other measured lifestyle factors, such as smoking and overweight/adiposity, which provides meaningful insight when considering the anti-cancer mechanism(s) of physical activity, discussed later in Parts 2 and 3.

### Effects of Regular Exercise on Tumour Growth: Key Findings From Rodent Models

A growing body of research has examined the effects of voluntary or forced exercise in rodents – performed before, during, or after a tumour challenge – on cancer growth, in comparison to non-exercising controls. Two systematic reviews have recently summarised rodent studies investigating the effects of exercise on tumour growth and metastasis (3, 4). The reader is directed to these publications for a comprehensive review of available studies. Here, for the convenience of readers, key studies demonstrating the anti-cancer effects of structured exercise training (e.g., forced treadmill running at a specific frequency, intensity, time, type (FITT) prescription) or physical activity (e.g., voluntary wheel running which is incidental and self-paced) in rodents are briefly summarised.

A landmark study showed that voluntary wheel running suppressed growth of cancers induced genetically, by carcinogen administration, and *via* cancer cell injection (24). For example, growth of subcutaneously injected B16 melanoma and Lewis lung carcinoma were suppressed by 61% and 58%, respectively, in mice that performed four weeks of daily wheel running prior to inoculation compared to mice without access to running wheels (24). Furthermore, daily wheel running performed for 11 months after intraperitoneal Diethylnitrosamine injection suppressed the development of liver tumours by 44% compared to mice without access to a running wheel (24). Finally, 20 weeks of voluntary wheel running tended to delay the development of malignant melanoma lesions in transgenic Tg(Grm1)Epv mice, compared to non-exercising controls (24).

Numerous rodent studies have employed models that involved injecting cancer cells. For example, ten weeks of treadmill running performed on five days/week – and commenced eight weeks prior to subcutaneous injection with Walker 256 carcinoma cells – suppressed tumour development by 91% and increased survival duration by 94%, compared to non-exercise controls (25). Other studies that also injected cancer cells and examined similar exercise regimens have replicated these findings (26–29). However, the immunogenicity of injecting foreign cancer cell lines into rodents may exaggerate the protective effects of exercise training on cancer growth, as discussed further in Part 3 of this review.

Another commonly used cancer model involves administering chemical carcinogens to rodents and measuring the development of cancers at sites that are sensitive to carcinogenesis caused by that chemical. For example, when voluntary wheel running performed for up to 3,000 wheel rotations/day was implemented for 52 weeks, with tumour challenge administered *via* daily feeding of 3’-Me-DAB from week 7-52, liver tumour incidence was 0% compared to 65% in non-exercising control rats (30). The protective effects of exercise against carcinogen-induced tumours have been shown in other studies that share similar methodologies (31–36).

Finally, a series of studies have demonstrated that the spontaneous development of tumours in rodents is suppressed by exercise. For example, Sprague Dawley rats that had access to a running wheel for 24 hours on alternate days from age 3-120 weeks, had lower incidence (38% *vs.* 54%) of pan-cancer, and tumour size was suppressed by 58%, compared to rats housed in small cages without access to a running wheel (37). A key design feature of this study was that it assessed the effects of exercise on spontaneous cancer development throughout the expected lifespan of Sprague Dawley rats, which is of clear relevance given the increased risk of cancer with advancing age in humans (38). Moreover, the assessment of spontaneous cancer development – rather than cancer induced by transplantation or by carcinogen-administration – closely reflects the development of many endogenous human cancers, and may therefore provide the most meaningful insight into the efficacy of physical activity in repressing cancer. Transgenic models provide a more targeted approach in discovering the effects of exercise on specific cancer types located in defined tissue types, and it is commonly reported that exercise elicits suppression of tumour growth when these models are used. For example, PTEN-deficient transgenic mice – which spontaneously develop liver cancers – that performed treadmill running for 32 weeks had lower incidence of detectable liver tumours (71% *vs.* 100%) and ~50% reduced tumour volume than non-exercising mice (39).

Overall, preclinical findings from rodent studies complement human epidemiology evidence and demonstrate that physical activity exerts anti-cancer effects leading to the suppression of cancer development. The mechanisms responsible for these effects remain subject to ongoing speculation, but as discussed next, we posit that altered immune regulation in physically active people is an integral mechanism, that leads to the prevention of cancer outgrowth.

## Part 2: Exercise-Induced Immuno-Modulation As An Integral Mechanism That Prevents Cancer Outgrowth

Despite the existence of robust preclinical and human epidemiology evidence indicating that physical activity commonly exerts anti-cancer effects, the mechanism(s) responsible for these outcomes remains ill-defined. Here, in Part 2, we explain why the reduced incidence of clinically diagnosed cancers among physically active individuals can be ascribed to modulation of immune regulation. To do this, we first begin by summarising the cancer immunoediting process – the most widely accepted contemporary theoretical model used to explain interactions between the immune system and cancer (8). We then outline how physical activity appears to modulate the path of the cancer immunoediting process. We highlight that altered immune-regulation – specifically to T cells – arising from regular exercise, enhances the elimination of cancer cell clones which have mutated to become immunogenic, predominantly culminating in the containment of cancers in a ‘precancerous’ or ‘covert’ equilibrium state. Whilst it has been discussed before that regular exercise could affect all stages of the immunoediting process (40), we emphasise here that the reduced incidence of cancers in physically active people may be ultimately derived from exercise-induced immuno-modulation which provides a final backstop preventing cancer outgrowth, thus delaying and/or averting a clinical diagnosis. Later in Part 2 we introduce the cancer immunogram – an established framework for understanding interactions between T cells and tumour cells (9) – and we explain why understanding this framework is of importance when seeking to understand how physical activity alters T cell regulation to reduce the incidence of clinically diagnosed cancers.

### Cancer Immunoediting and Its Relevance to the Anti-Cancer Mechanism of Physical Activity

Cancer immunoediting describes phases of interaction between cancer and the immune system that determine the presentation of clinically diagnosed cancer (8, 41, 42). The first ‘elimination’ phase describes the deletion of immunogenic cancer cells *via* a coordinated immune response. The anti-cancer immune response represents a cyclical, self-propagating process known as the ‘cancer immunity cycle’ (43). The cycle is initiated by the recognition of cancer antigens by antigen-presenting cells, such as dendritic cells. Cancer antigens can be broadly grouped as 1) ‘non-self’ or ‘tumour-specific’ antigens expressed only by cancer cells (i.e., mutation-derived neoantigens and onco-viral antigens), or 2) ‘self’ or ‘tumour-associated’ antigens that are expressed on/in healthy cells and overexpressed in cancer cells (e.g., cancer-germline antigens and cell lineage differentiation antigens) (44, 45). A key difference between tumour-specific and tumour-associated antigens is that tumour-specific antigens – neoantigens and onco-viral antigens – are more immunogenic, due to the central and peripheral immune tolerance mechanisms that limit autoimmunity by dampening immune responses to self-antigens (46).

Following antigen encounter, dendritic cells home to lymph nodes and present cancer antigen to activate T cells, which traffic through the circulation and infiltrate tumours (43). Within the tumour microenvironment, T cells recognise cancer antigens (e.g., neoantigens) displayed on major-histocompatibility complex (MHC)-1 expressed by cancer cells by binding *via* their T cell receptors, leading to T cell killing of cancer cell targets. The cancer-immunity cycle completes when cancer antigens are released upon cancer cell death, increasing the depth of response in subsequent cycles (43). In addition to anti-tumour T cell responses, natural killer (NK) cells elicit cytotoxicity against cancer cells with low MHC-1 expression (known as the ‘missing self’) due to the removal of inhibitory signalling between MHC-1 and killer immunoglobulin-like receptors on NK cells (47). Although, MHC-1 downregulation tends to occur in the more advanced stages of cancer progression (48) and thus NK cells do not predominate in early stage tumours (49). A successful elimination phase of cancer immunoediting prevents the establishment of clinical cancer. However, the persistence of less immunogenic cancer cells that evade immune elimination results in transition to an ‘equilibrium’ phase, whereby immunogenic cancer cells are removed but cancer cells with low immunogenicity persist (8, 41, 42). Low immunogenicity is characterised by defective antigen expression by cancer cells or defective presentation by antigen-presenting cells (e.g., reduced antigen processing and presentation due to suboptimal activation) (50). In equilibrium, a dormant tumour of stable size but with selective growth advantages is contained by the immune system (8, 41, 42). Maintenance of the dormant tumour within the equilibrium phase is reliant on adaptive immunity – and specifically CD8^+^ T cells – as opposed to innate cells such as NK cells (42) as the large majority of cancers in the equilibrium phase of immunoediting express MHC-1 (48). The equilibrium phase may be sub-clinical, and as such, maintaining cancers in this phase contributes to a reduced risk of clinical cancer diagnosis. Cancers ‘escape’ immune control due to a failure in the ability of T cells to identify or eradicate the cancer cells which have progressively been immunologically-sculpted, and as a consequence grow unrestrained by immune pressure, resulting in clinical cancer (8, 41, 42). We discuss later in Part 3 how physical activity might augment the ability of CD8^+^ T cells to identify and/or eradicate tumour cells leading to the containment of cancer outgrowth.

Consideration of the cancer immunoediting process is of fundamental importance when considering how physical activity reduces the incidence of clinically-diagnosed cancers. As we outline next, there is a lack of human evidence showing that physical activity ‘prevents’ the initiation and early promotion of cancer. This is important because it has been hypothesised that physical activity ameliorates a multitude of different endogenous factors – such as sex hormones, oxidative stress, inflammation, and other factors (7) – that are otherwise thought to be responsible for initiating/promoting cancer growth. However, the lack of human evidence that physical activity prevents early neoplasia raises questions about whether physical activity sufficiently ameliorates factors that drive cancer initiation, and rather this emphasises that physical activity instead helps to prevent the later stages of cancer outgrowth. This hints that the adaptive immune system – and specifically T cells, which are ultimately responsible for controlling cancer outgrowth (42) – should be considered as a mechanism-of-action linking physical activity level to reduced risk of a clinical cancer diagnosis. This theory is not at odds with other theories which suggest that physical activity ameliorates endogenous factors that directly stimulate cancer (7), and indeed that may still be the case. Rather, this theory suggests – even if exercise does suppress different endogenous factors that stimulate cancer initiation – that immune regulation is also augmented by physical activity and ultimately this provides a final backstop to counter abnormal cell outgrowth.

#### Evidence That Physical Activity Does Not Prevent Early Neoplasia but Instead Helps to Prevent Cancer Outgrowth

Here we explain that there is evidence showing that physical activity does not ‘prevent’ the early stages of cancer development (e.g., initiation and initial promotion), and instead physical activity appears to yield greater efficacy against the later stages of disease (e.g., progression to more advanced stages of cancer).

Before summarising evidence to support this inference, it is important to explain the premise that every cancer arises from a subclinical precursor state, and that these precursor cancers are less immunogenic than more advanced stages of that same cancer. Numerous detectable precursors to clinical cancer exist, and include: monoclonal gammopathy of undetermined significance and smouldering multiple myeloma (precursors to multiple myeloma); monoclonal B cell lymphocytosis [precursor to chronic lymphocytic leukaemia (CLL)]; cervical intraepithelial neoplasia (precursor to cervical cancer); polyps of the gallbladder or colon (precursors to gallbladder and colon cancer, respectively), and numerous others. As is the case in the aforementioned examples, it is theorised that every cancer develops in a step-wise manner from a precursor state with very few mutations, and these precursors are undetectable in almost all tissue sites until further mutations occur (51). In support, previously undetected or ‘covert’ cancers of the breast, prostate, lung, and thyroid have been identified incidentally at autopsy in individuals without a history of related disease (52–55). Furthermore, ovarian cancers have been identified during benign surgeries to remove fallopian tubes in women without an existing history or hereditary risk factors for ovarian cancer (56, 57).

It is often speculated that physical activity ‘prevents’ spontaneous cancer from arising. However, there is evidence that physical activity does not stop cancer initiation from arising in the first place, and rather it appears that physical activity ultimately averts progression to more advanced cancer. This is captured by epidemiology studies assessing the risk of clinically diagnosed cancers – and their related precursors – according to physical activity level. Cross-sectional research shows that the risk of cancer precursors is less affected by physical activity, but the risk of related clinical cancer is reduced in physically-active individuals. For example, a higher physical activity level reduces the risk of oesophageal adenocarcinoma (2) but not Barrett’s oesophagus (58, 59). Similarly, a higher physical activity level was not associated with the development of small colon adenomas, yet reduced the risk of large adenomas and colon cancer (60). Furthermore, the risk of lymphocytic leukaemia is not reduced in those with a higher physical activity level (2) which is likely explained by a high proportion of CLL cases, which is commonly indolent, asymptomatic, and not actively treated, akin to cancer precursor conditions (61, 62).

In support of the notion that physical activity reduces clinical cancer risk by averting cancer outgrowth (i.e., the transition from equilibrium to escape), it was shown in a randomised-controlled trial where a holistic lifestyle intervention, including exercise training, was implemented for one year during active surveillance for early-stage prostate cancer that there was a reduced likelihood of disease reclassification in the intervention group, but no change to prostate specific antigen (PSA) (63) or a modest (−4% ≈ coefficient of variation (CV) 2.9%^2^) reduction (64) within the biological variation for PSA, which has been estimated at CV ~20% (65–67) or more recently CV ~7% (68). Similarly, recent results from a randomised-controlled trial of 12 weeks high intensity interval training *vs.* usual care in men undergoing active surveillance for prostate cancer revealed a statistically significant between-group difference in PSA (−1.1 μg/L, *P*=.04) but the reduction from baseline in the exercise group was within biological variation of PSA (−6.6% ≈ CV 4.8%^2^) (69). It has also been shown in other studies that physical activity level is associated with lower risk of disease reclassification in men undergoing active surveillance for low-grade prostate cancer (70, 71). Together, using prostate cancer as an example, it appears that physical activity or exercise training does not eliminate or reverse early-stage cancer – e.g., in this case, those cells overtly producing PSA – but instead may sustain the maintenance of precancers in equilibrium.

On the other hand, evidence from animal studies appears, at first, to support the notion that physical activity does prevent spontaneous cancers from initiating. For example, in a study that assessed spontaneous cancer incidence over the lifetime of Sprague Dawley rats, it was found that spontaneous cancers developed in 38% of exercising animals *vs.* 54% in non-exercising animals (37). However, as the tumours in this study were detected by palpation, it may be the case that dormant precursors were undetected in the exercising animals. Indeed, in animal studies where transgenic models have been used, which permits more targeted induction of tumours at specific anatomical locations, evidence indicates that regular exercise does not consistently stop spontaneous cancer initiation, but rather, tends to delay and/or avert the progression of smaller cancers to more advanced cancer (24, 72). For example, in a study comparing the effects of regular exercise *vs.* no-exercise in transgenic Apc^Min/+^ mice, the number of colon polyps was not different between groups, but the number of large polyps was reduced in exercising mice (72). On the other hand, another comprehensive study (73), employing moderate exercise in nfkb1^-/-^ mice – which is used as a model for assessing disease and cancer in the liver – found that exercise almost entirely averted the development of detectable neoplasia, in a mechanistic process thought to be dictated, at least in part, by amelioration of inflammation. This study suggests that regular exercise may in fact avert early neoplasia, in a process that may be independent of immune involvement (73). However, it was not practically feasible, due to the lack of cancer development in those animals, to infer immune involvement in this process. Given the reduced frequency of immune cells in the local tissue of these animals after exposure to regular exercise, it may be that exercise in this case averted cancer by ameliorating inflammation – and that the immune system was not needed to prevent cancer cell outgrowth – or, that the immune system was involved in eliminating neoplasia at an undetectable level (73).

When considering how physical activity averts cancer outgrowth, an important point is that not all cancers are ‘spontaneously’ induced, and can instead be initiated by other factors including carcinogen exposure. Importantly, animal studies appear to indicate that regular exercise can exert anti-cancer effects against tumours initiated by carcinogen exposure (24, 30–36). For example, regular exercise for 11 months after intraperitoneal Diethylnitrosamine injection suppressed the development of liver tumours by 44% compared to mice without access to a running wheel (24). Given such findings, where the mode of cancer induction – *via* acute, high-dose carcinogen exposure – likely precludes the prevention of cancerous DNA mutations, then clearly exercise is able to elicit anti-cancer effects after cancer initiation to avert progression.

In seeking to identify the mechanistic factor(s) responsible for countering cancer outgrowth, it is important to consider – as previously outlined – that physical activity is associated with a reduction to a diverse array of clinically diagnosed cancers (2). Thus, the mechanism induced by physical activity that suppresses cancer outgrowth must be applicable to an array of cancers which are highly heterogenous – for example, comprising entirely different tissue types (e.g., carcinoma *vs.* lymphoma), located between different tumour microenvironments (e.g., solid *vs.* liquid tumours), exhibiting profoundly different cellular structures (e.g., expressing different cell surface signalling receptors, such as sex hormone receptors) and harbouring different genetic and epigenetic features (e.g., between cancer subclones). The only biological entity capable of countering such heterogeneity is the immune system. More specifically, as outlined earlier in this section, and discussed in more detail next, if a dormant/covert pre-cancer has successfully been established – induced spontaneously or otherwise – the immune system is principally reliant on adaptive immunity, and specifically T cells, to prevent cancer outgrowth and maintain the cancer in equilibrium (42).

#### T Cell Immunogenicity May Be Fundamental to the Mechanism-of-Action Linking Physical Activity With the Prevention of Cancer Outgrowth

A principal feature of cancer precursors is a lack of immunogenicity which permits ongoing evasion from the immune system, supporting survival. Indeed, immunogenicity of the cancer cell clone is pivotal to successful elimination of cancerous cells by the immune system, as outlined in the cancer immunoediting process (8, 41, 42). Importantly, a key feature of preclinical cancers, as compared to clinically diagnosed or late-stage cancers of the same tissue type, is their relatively low mutational burden, which is associated with relatively low immunogenicity to T cells. Tumour mutational burden regulates the expression of tumour neoantigens that determine immunogenicity, as somatic mutations generate neoantigen peptides that are expressed *via* MHC-1 on the cancer cell surface and recognised as foreign by host CD8^+^ T cells (46); this process does not involve NK cells which, as outlined earlier, identify foreignness *via* lack of MHC-1 (i.e., ‘missing self’). As examples, prior sequencing studies have identified reduced mutational burden for Barrett’s oesophagus compared with oesophageal adenocarcinoma (74) and colon adenoma compared with colon adenocarcinoma (75). Furthermore, in another example, tumour mutational burden in prostate cancer increases with tumour stage and lymph node involvement (76). Therefore, in the event of further mutations arising in damaged or cancerous cells, these cells become more immunogenic *via* expression of neoantigens, and may either (i) be eliminated by T cells in hosts with sufficient immune competency to maintain the cancer precursor in covert equilibrium, or (ii) not be eliminated by T cells in hosts with insufficient immune competency resulting in more accelerated outgrowth of clinical cancer.

**Figure 1:**
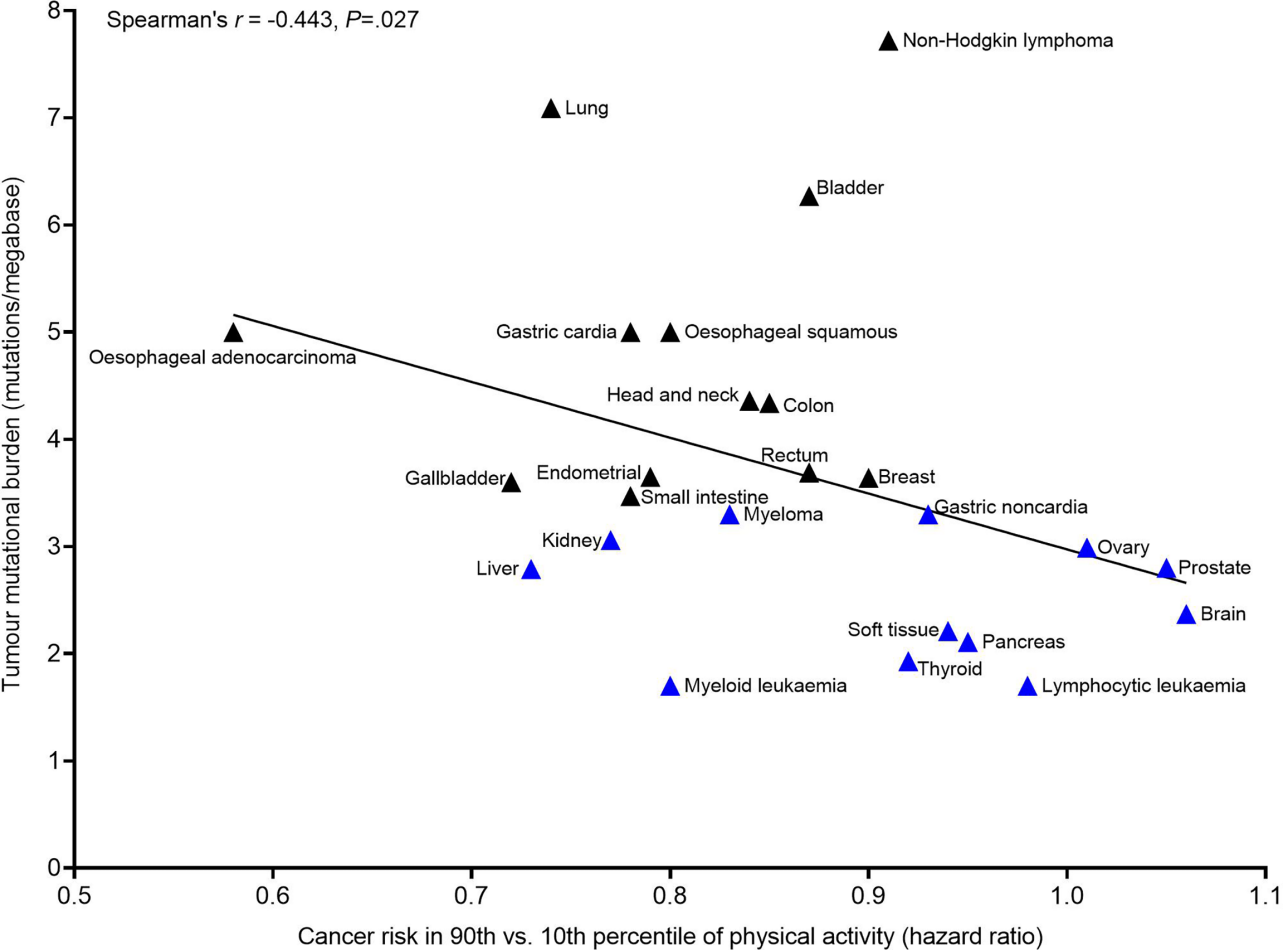
Association between the tumour mutational burden (TMB) of a cancer tissue location and the magnitude of risk reduction for that cancer site in the 90^th^ *vs.* 10^th^ percentile of self-report leisure-time physical activity. Cancer risk in the 90^th^ *vs.* 10^th^ percentile of physical activity data were obtained from ref (2). TMB data were obtained from ref (77). Blue triangles indicate cancer sites with a TMB below the median (<3.47 mutations per megabase) and black triangles indicate cancer sites with a TMB equal to or above the median (≥3.47 mutations per megabase). Associations between TMB and cancer risk in 90^th^ *vs.* 10^th^ percentile of physical activity were examined using Spearman’s r as data were not normally distributed (Shapiro-Wilk P>.05). Analysis was performed in Graph Pad Prizm v9.0.1 (GraphPad Software, California, USA). Melanoma was excluded from analysis due to established confounding effects of UV exposure on cancer risk associated with physical activity. TMB data from ref (77) included multiple different cancer subtypes within a given tissue location. The TMB of relevant cancer subtypes was calculated for each of the cancers reported in ref (2), guided by the International Classification of Diseases for Oncology 3rd Edition, as per Supplemental Table 3 in ref (2). This calculation was adjusted to control for the frequency of samples (per cancer subtype) analysed in ref (77), in an attempt to adjust for the relative incidence of different cancer subtypes among the general population. Nevertheless, a limitation of this approach remains an assumption that different cancer subtypes – which commonly varied in TMB – were equally represented in the sampling of ref (2) and ref (77).

If physical activity does regulate T cells in a manner that promotes the elimination of cancer cells exhibiting more overt immunogenicity – and if this is the overarching mechanism elicited by physical activity that prevents cancer outgrowth – then evidence of this should be apparent in human epidemiology studies, due to inherent variation in the mutational (i.e., neoantigen) burden of different cancers, and variation in the degree of efficacy of physical activity in reducing clinical cancer cases. Indeed, it appears that the mutational burden of cancers located at different tissue locations may predict the magnitude of risk reduction in physically active individuals. In the largest analysis to date, hazard ratios for clinical cancer risk (due to high *vs.* low physical activity levels) at different tissue sites demonstrated a high degree of variability (hazard ratio = 0.58 to 1.27) (2). Here, using previously published data we show the potential relevance of T cell immunogenicity to these aforementioned findings (**Figure 1**). Specifically, we paired hazard ratios for clinical cancer risk (due to high *vs.* low physical activity levels) at specific anatomical sites (2) together with previously published tumour mutational burden data for those cancer sites (77). In combining these data, a negative correlation between tumour mutational burden and hazard ratio for risk of clinical cancer in physically active individuals is apparent (Spearman’s *r* = −0.443, *P*=.027). This suggests a greater risk reduction *via* physical activity for cancers which typically exhibit a higher tumour mutational burden, and little benefit of physical activity against cancers which typically exhibit a low mutational burden. This implies that an advanced number of mutations (and neoantigens) might be needed for the effects of physical activity to manifest in human epidemiology studies. At the same time, in cancers with a low mutational burden (and a lower likelihood of expressing neoantigens) there is no beneficial effect of physical activity apparent in human epidemiology studies. This strongly positions the immune system in the anti-cancer effects of physical activity, as an integral mechanism preventing cancer outgrowth.

It is important to note that tumour mutational burden is an imperfect model of T cell immunogenicity, because not all mutations produce neoantigens (78, 79), thus it may be the case that neoantigen burden better predicts the efficacy of physical activity than tumour mutational burden alone. For example, some cancers – such as acute myeloid leukaemia – are thought to require very few somatic mutations to produce high quality neoantigens that provoke strong immune responses (77, 80–82), therefore it may be that the seemingly beneficial effects of physical activity in reducing the incidence of those clinically diagnosed cancers are explained by neoantigen expression despite having a relatively low mutational burden (2). It is also important to acknowledge that a high neoantigen burden on its own does not guarantee strong T cell responses. Indeed, we outline next how an array of factors dictate T cell success against tumour cells, as predicted by the cancer immunogram (9). In doing so, we highlight that each of these factors should be considered when seeking to understand how physical activity might help to avert cancer outgrowth.

#### The Cancer Immunogram – A Framework for Understanding How Physical Activity Regulates T Cell Responses Against Tumour Cells

In recent years, the cancer immunogram has been developed as a framework to predict the success of cancer cell elimination by T cells (9). The cancer immunogram identifies seven broad factors that determine T cell success against tumour cells: (i) tumour ‘foreignness’ to T cells; (ii) tumour ‘visibility’ to T cells; (iii) general T cell status in blood; (iv) T cell infiltration to tissues; (v) presence of immune checkpoints associated with T cell exhaustion and anergy; (vi) presence of inflammatory inhibitors of T cells; and (vii) presence of metabolic inhibitors of T cells (9).

Two of the aforementioned immunogram factors can be considered tumour-intrinsic determinants of T cell success: tumour ‘foreignness’ and tumour ‘visibility’. Tumour ‘foreignness’ to T cells is dictated by somatic mutations which give rise to neoantigen expression to T cells, and as outlined earlier, it appears that cancers which have a higher likelihood of expressing neoantigens demonstrate the greatest sensitivity to physical activity. The ‘visibility’ of foreign tumour cells to T cells is also critical in dictating the success of T cell responses. MHC-1 expression by tumour cells enables neoantigen peptide recognition by CD8^+^ T cells. However, whilst MHC-1 is omnipresent in the early stages of most cancers (48), it is often lost as cancers progress (83) which removes the ability of CD8^+^ T cells to recognise cell surface neoantigens (84). Therefore, cancers with a high neoantigen burden, but dysfunctional MHC-1 expression, may evade elimination by CD8^+^ T cells due to the inability of CD8^+^ T cells to ‘visualise’ their ‘foreignness’. This loss of MHC-1 may explain why some cancers – for example, non-Hodgkin lymphoma (85) (of which, diffuse large B cell lymphoma is the most common) – exhibit less sensitivity to physical activity (2) despite commonly exhibiting a high tumour mutational burden (77) (**Figure 1**). Interestingly, in such a scenario, NK cells – rather than CD8^+^ T cells – would be expected to predominate the immune response preventing cancer outgrowth, and we speculate that NK cells are less involved in the anti-cancer effects of physical activity – this is discussed more in Part 3.

To the authors’ knowledge, no studies have assessed the direct effects of regular exercise on the speed of tumour cell neoantigen acquisition, nor loss of MHC-1. As previously mentioned, it is commonly theorised in exercise oncology that physical activity reduces cancer growth by ameliorating endogenous stimulants of cancer initiation/promotion – such as sex hormones, oxidative stress, inflammation, and other endogenous factors (7). Such a mechanism could hypothetically slow or avert cell division/damage leading to a delay in the acquisition of mutations. Whilst this may indeed be the case, it is not convincing that exercise-induced reductions to such endogenous factors (that otherwise directly stimulate cancer growth) act alone to avert the development of cancers. For example, it is unlikely that the amelioration of a single endogenous cancer stimulant (e.g., a sex hormone), which affects only a limited number of cell types (e.g., sex hormone sensitive cancers), explains why physical activity averts cancer outgrowth across so many different tissues. On the other hand, if it were the case that physical activity ameliorates endogenous cancer stimulants that have the capacity to directly initiate/promote cancer to cells located across a diverse array of tissues (e.g., inflammatory mediators), then it would be expected that the incidence of *all* spontaneous cancer types (i.e., across all tissue types) would be decreased in physically active *vs.* inactive people. This does not appear to be the case, as only some cancers exhibit reduced incidence in highly active people, whereas others do not (2). For example, exercise ameliorates biomarkers indicative of brain inflammation (86), but physical activity is not associated with a reduced incidence of brain cancers (2). Moreover, if it were true that physical activity nullifies cancer growth – in isolation – by ameliorating tumour growth directly, then it might be expected that physical activity would reduce the incidence of cancers which are linked to a high lifetime frequency of cell divisions to healthy progenitor cells, which concomitantly gives rise to an associated increased chance of cancerous mutations arising (87, 88). However, evidence suggests that physical activity does not consistently reduce the incidence of such cancers. For example, pancreatic cancer and CLL, which are examples of cancers linked to a high lifetime frequency of cell divisions to healthy progenitor cells (87, 88), do not consistently show reduced incidence in people who report high levels of physical activity (2). Thus, it would seem that healthy cells from different anatomical sites which undergo the largest frequencies of cell divisions do not have a consistently reduced risk of developing into cancer in physically active people, whom would purportedly have ameliorated levels of endogenous factors that directly cause cell growth; further research is warranted to confirm this observation. Separately, it is worth considering that if the aforementioned was true, and regular physical activity induces anti-cancer effects solely by ameliorating tumour growth factors, it might be expected that commencement of regular exercise training (in those previously considered physically inactive) would lead to a substantial reversal of cancer growth (by lowering endogenous stimulants e.g., inflammatory mediators). We are not aware of any convincing evidence that this happens. Lastly, we remind the reader that human epidemiology evidence illustrates that mutations might be needed to permit the anti-cancer effects of physical activity to manifest (**Figure 1**). Thus, together, these inferences collectively indicate that even if physical activity ameliorates factors that directly simulate cancer growth, and even if there is a concomitant reduction to the acquisition of carcinogenic mutations, T cells are likely to be involved as a backstop, leading to the elimination of cancer outgrowth and maintenance of cancers in equilibrium.

Accordingly, we assume that – for physical activity to enhance the elimination of tumour cells – the cancer needs to be immunogenic, i.e., both foreign (e.g., expressing neoantigens) and visible (i.e., expressing MHC-1) to T cells. Next in Part 3, we focus our attention on assessing how physical activity might alter T cell regulation, to enhance the elimination of immunogenic tumour cells. To do this, we evaluate the role of physical activity on the five remaining factors of the cancer immunogram (9). The five remaining determinants can be considered T cell-intrinsic, and a growing body of literature predicts that each of these determinants can be altered by regular exercise. We anticipate that understanding the effects of physical activity on these five determinants of T cell function will help foster better understanding of how physical activity averts cancer outgrowth.

#### Conclusion

In Part 2, we highlighted that human epidemiology evidence shows that physical activity does not appear to ‘prevent’ the initiation or early promotion of cancer, but rather, it appears to be more effective in delaying progression of early neoplasia (i.e., benign/dormant precursors) to more advanced cancers. Thus, it may be that physical activity does not prevent cancer, but instead maintains cancers in equilibrium, thus delaying/averting a clinical cancer diagnosis. We also demonstrated that a shared feature of the clinically diagnosed cancers which have a reduced incidence among physically active people is their more overt immunogenicity, which appears to be linked to a higher mutational (i.e., neoantigen) burden. This strongly implicates the immune system – and specifically T cells – as a major contributor to the anti-cancer mechanisms of physical activity. The possible reasons explaining how regular exercise modifies T cells to eliminate immunogenic cancer cell outgrowth is discussed next in Part 3.

## Part 3: Evaluating the Effects of Physical Activity on T-Cell Intrinsic Factors Within the Cancer Immunogram

Evidence summarised in Part 2 implicates the immune system – and specifically T cells – as a major contributor to the anti-cancer mechanisms of physical activity. Therefore, in Part 3, we consider how physical activity might regulate T cell function to augment effector competency against immunogenic cancer cells. To do this, we evaluate the effects of physical activity on five T cell-intrinsic factors that determine the successful elimination of immunogenic tumour cells, as outlined in the cancer immunogram (9), these are: (i) general T cell status in blood, (ii) T cell infiltration to tissues, (iii) presence of immune checkpoints associated with T cell exhaustion and anergy, (iv) presence of inflammatory inhibitors of T cells and (v) presence of metabolic inhibitors of T cells. After evaluating available evidence, we then synthesise how physical activity-induced changes to these determinants may individually - or in combination - explain how physical activity maintains cancers in equilibrium.

### Determinant 1 – Physical Activity Alters General T Cell Status in Blood

The first T cell-intrinsic determinant within the cancer immunogram is general T cell status in blood (9). To date, research assessing the effects of physical activity on general T cell immune status in blood has largely centred on immunosenescence. Immunosenescence describes age-associated dysfunction within the innate and adaptive immune system that compromises host protection against infectious, inflammatory, autoimmune, and neoplastic conditions (89, 90). Central to prior exercise immunology research in this area is the theory that lifelong physical activity ameliorates features of immunosenescence. The relevance of this concept appears to be reinforced by the observation that the many cancers which appear to exhibit lower incidence among physically active people – as compared to less physically active people – are cancers that arise in older age (2). Moreover, there is a paucity of evidence that physical activity reduces the incidence of paediatric cancers. Indeed, established reviews summarising the environmental risk factors for childhood cancer do not acknowledge inadequate physical activity as a risk factor (91, 92). Additionally, in a previously described rodent study assessing spontaneous cancer development over the lifetime of Sprague Dawley rats, it was observed that regular exercise had no effect on cancer incidence until the age of 85 weeks (37), which approximates to an age >60 years old in humans. The aforementioned examples imply that physical activity may be altering the process of immunosenescence, leading to reduced clinical cancer risk. However, we caution that this may not be the case *per se*. The mutational burden of different cancers increases with ageing (77), and so if physical activity is augmenting T cell responses against immunogenic tumour cells (e.g., those that are highly mutated), it is not necessary to rely on another ageing-induced factor – such as immunosenescence – to induce these benefits. As outlined in Part 2, cancers arising in paediatric populations, including acute lymphoblastic leukaemia, produce high quality neoantigens that provoke strong immune responses, and so the potential anti-cancer benefits of physical activity should not be discounted in younger people; this requires confirmation in future studies.

In considering the potential anti-immunosenescence effects of physical activity in the context of cancer risk here in Determinant 1 it is important to consider, as outlined in Part 2, that it is predominantly T cells – rather than NK cells – that are necessary to maintain cancers in equilibrium (42) and thus the immunological focus of this section is principally on the adaptive immune system, specifically T cells. Changes to innate immune cells, such as NK cells, with advancing age has been reviewed previously (93, 94). Additionally, NK cells turnover within 14 days (95) whereas the estimated half-life of memory T cells is ~200 days and naïve T cells is ~2,000 days (96). Thus, NK cells are more commonly investigated in the context of acute short-term exposure to exercise over minutes and hours (see *Determinant 2*), rather than habitual physical activity or regular exercise training over months and years – as will be discussed here in Determinant 1.

Features of T cell immunosenescence include proportional increases in late-differentiated ‘senescent’ memory T cells with concomitant decreases in the proportion of naïve T cells, which are thought to be driven by prior/latent infections (e.g., *Cytomegalovirus*, CMV), thymic involution, and other age-associated factors (97, 98). T cell immunosenescence has been associated with increased clinical cancer risk, due to a reduced capacity to respond to novel cancer neoantigens because dysfunctional senescent T cells have accumulated, and naïve T cell output is compromised (99). Central to Determinant 1, is the idea that physical activity may reduce the accumulation of senescent T cells and/or maintain naïve T cell frequency (100–102), which may in turn preserve the capacity of T cells to respond to neoantigens (99). The relative effects of physical activity on senescent T cells and naïve T cells are summarised and evaluated in turn next.

#### Physical Activity and ‘Senescent’ T Cells

It is widely considered that a physically active lifestyle ameliorates the accumulated frequency of circulating senescent T cells. Different theories exist to explain how physical activity may exert this potentially beneficial effect (99–101). For example, one hypothesis predicts that physical activity may limit the accumulation of senescent T cells by augmenting immune competency leading to a reduction in CMV reactivation (which is common among the general population) (99). Another prominent hypothesis – called the ‘immune space’ theory – predicts that repeated acute exposure to exercise drives reductions in the number of senescent T cells. This hypothesis is characterised by three distinct phases (100, 101). Firstly, senescent T cells are preferentially mobilised in response to epinephrine, which increases during acute exercise, and these cells are then redistributed to peripheral tissues to conduct immunosurveillance (discussed more in Determinant 2). Secondly, after conducting immunosurveillance, senescent T cells undergo apoptosis in peripheral tissues upon encounter with pro-apoptotic signals. Thirdly, apoptosis of senescent T cells is hypothesised to create ‘immune space’ for the generation of new naïve T cells *via* a negative-feedback loop (100, 101). A criticism of this hypothesis which has been highlighted is the purported fixed-size immune system (103), though there is animal evidence showing that experimentally induced lymphopenia, and the subsequent ‘open space’, drives naïve T cell proliferation, in a process termed lymphopenia-induced proliferation (104). However, this process may largely result in memory T cells against self-antigens (105). Other tenets of the immune space hypothesis have been disputed, including the susceptibility of senescent cells to apoptosis, and the perceived advantage of selectively deleting senescent T cells that are required to maintain active immune control (103). Indeed, clonal senescent T cells appear to confer a prognostic advantage in at least some cancers (106, 107) – even though, as previously stated, it is thought that senescent T cells might be less functionally competent.

When considering the hypotheses above, it must be considered that evidence linking physical activity with reductions to archetypal features of T cell senescence – for example, using the cell surface markers CD57 and/or KLRG1, or telomere length – is inconsistent. In one study, males with higher cardiorespiratory fitness had lower proportions of senescent CD4^+^ and CD8^+^ (CD57^+^KLRG1^+^) T cells compared to those with lower fitness (108). However, no differences were observed in senescent CD4^+^ or CD8^+^ (CD57^+^) T cells in a cross-sectional study comparing master’s cyclists aged 55-79 years (with a 21-year training history) and less active healthy age-matched counterparts (109). Furthermore, a review of trials assessing the role of physical activity on immunosenescence determined *via* telomere length revealed inconsistent findings (110). For example, in one trial, a higher self-reported physical activity level was associated with longer telomeres in blood leukocytes (111), yet another trial reported no association between self-reported physical activity level and blood leukocyte telomere length (112).

A paucity of randomised-controlled intervention trials have been conducted to investigate the effects of exercise training on senescent T cells, as reviewed elsewhere (110, 113). One randomised-controlled trial showed that a six-week resistance training programme reduced the frequency of senescent CD8^+^ and CD8^−^ (CD57^+^CD28^−^) T cells in blood from baseline in CMV-seropositive females aged >65 years, but the change was not different to that observed in the non-exercising control group (114). Furthermore, resistance training did not reduce senescent T cell frequency in blood in CMV-seronegative individuals, despite the presence of CD8^+^CD57^+^CD28^−^ T cells in blood at baseline, albeit at lower frequencies than CMV-seropositive individuals (~5 cells/μL *vs.* ~26 cells/μL) (114). Another randomised-controlled exercise intervention study showed that aerobic exercise training performed in hypoxic (15% oxygen) but not normoxic (21% oxygen) conditions reduced the frequency of senescent CD4^+^ (KLRG1^+^) T cells in blood from baseline, but comparisons to non-exercising control groups were not reported (115). Furthermore, a longitudinal observational study – employing regular follow-up of triathletes during a six-month training period – revealed no change to the proportions of senescent CD4^+^ and CD8^+^ (CD57^+^KLRG1^+^) T cells when compared with pre-training cell proportions (116). Finally, telomere length was unchanged in blood leukocytes of middle-aged women following 6 months (117) and 12 months (118) of aerobic exercise training compared to non-exercising control groups. It is questionable therefore whether physical activity reduces the frequency of archetypal senescent T cells in blood. However, inconsistencies in study design, and limitations in methodologies – such as inadequate control for CMV serostatus in many studies – precludes any firm conclusions being made. Nevertheless, there are other aspects of the T cell ageing process which may be altered by physical activity, as discussed next.

#### Physical Activity and ‘Naïve’ T Cells

Alongside purported changes to senescent T cells discussed above, it is widely considered that lifelong physical activity may lead to better maintenance of circulating naïve T cell frequencies in older adults. Increased naïve T cells due to physical activity may explain reductions to clinical cancer risk, as a diverse array of naïve T cells are required to detect novel cancer neoantigens arising, which are increasingly expressed by cancer cells as they accumulate mutations and become immunogenic (119). Indeed, declines in T cell production due to thymic involution has been stated as a primary cause of cancers (120), and reduced T cell receptor diversity is associated with worse prognosis in certain cancers when compared to a more diverse T cell repertoire (121, 122).

There are a number of prominent studies which appear to show support for the theory that physical activity promotes naïve T cell frequency in blood. In one aforementioned study, males with higher cardiorespiratory fitness had higher proportions of naïve CD8^+^ (CD28^+^KLRG1^−^) T cells than male counterparts with lower fitness (108). Consistent with these observations are the findings from another aforementioned study comparing master’s cyclists to less active healthy age-matched counterparts (109). In that study, physically active older adults showed higher proportions of blood naïve (CD45RA^+^) CD4^+^ and CD8^+^ T cells compared to less active age-matched counterparts. However, other studies that reported naïve T cells using more comprehensive gating strategies – using CD45RA in conjunction with CCR7, CD27 and/or CD28, which better enables assessment of T cell differentiation (123) – found no differences in naïve T cell proportions between groups of adults aged >50 years with an athletic training history *vs.* healthy but less active age-matched counterparts (124–126), or increased CD4^+^ naïve T cell proportion but reduced CD8^+^ naïve T cell proportion in adults aged >60 years undertaking >10,500 *vs.* <5,000 steps/day (127).

Inconsistencies from cross-sectional studies are shared in studies assessing changes to the proportion/frequency of naïve T cells from pre- to post-exercise training. Indeed, exercise intervention trials report increases (114, 128–130) or no change (131–134) to naïve T cells following exercise training (duration: 3 weeks to 12 months). As with cross-sectional evidence, there are inconsistencies in immunophenotyping strategies used to identify naïve T cells. Indeed, the majority of studies used either CD45RA^+^ (131, 133, 134) or CD28^+^ (114, 129, 130, 132) alone to identify naïve T cells in blood. In one study which identified naïve T cells, using more advanced phenotyping, as CD45RO^−^CCR7^+^, it was found that exercise training increased the proportion of CD8^+^ naïve T cells in blood (128); this study also reported a proportional decline in late differentiated CD45RO^−^CCR7^−^ effector memory T cells and increases in CD45RO^+^CCR7^+^ central memory cells (128); it is unclear whether the proportional changes to T cell memory subsets are an artefact of reciprocal changes within compositional data. Taken together, it can be concluded from the balance of evidence that physical activity may increase the frequency/proportion of T cells in the blood with a phenotype akin to naïve T cells, but this is not a consistent finding. Discrepant findings among prior studies are likely due to differences in study design, population, and analytical methodologies used.

#### Possible Mechanisms for a Higher Frequency of ‘Naïve’ T Cells in Physically Active People

Different theories exist in the literature to explain why an active lifestyle may be associated with higher circulating naïve T cells (100–102). For example, the previously described ‘immune space’ hypothesis predicts that senescent T cells undergo apoptosis in response to acute bouts of exercise, leading to the creation of ‘immune space’ for the generation of new naïve T cells *via* a negative-feedback loop (100, 101). As outlined earlier, reductions to senescent T cells – characterised by archetypic cell surface markers CD57 and KLRG1 – are not consistently seen in physically active persons. Thus, other theories which may explain the increase in naïve T cell frequency among physically active individuals, and which do not comprise the deletion of potentially valuable cancer-specific senescent memory T cells, are considered.

An important series of studies have shown that physically active individuals exhibit higher concentrations of immunoregulatory cytokines, such as interleukin (IL)-7 and IL-15 in blood (109, 127), which may be due to increased homeostatic cytokine output from skeletal muscle (102, 108) as both IL-15 and IL-7 are expressed in skeletal muscle (135, 136). It is unclear whether acute, contraction-induced elevations to IL-7 and IL-15 following aerobic (137) or resistance (138) exercise, and/or adaptive training responses such as muscle hypertrophy or change to muscle fibre-type composition, underpin elevated blood IL-7 and IL-15 in physically active individuals, and further research is needed. It has been hypothesised that IL-7 and IL-15 secretion from muscle may lead to better maintenance of naïve T cell output from the thymus by increasing the amount of cells entering and exiting the thymus (99, 102). However, it is unclear if this is the case. For example, it seems that CD34^+^CD10^+^ cells (considered thymic immigrants) do not appear to be strongly influenced by physical activity (109). Additionally, CD4^+^PKT7^+^ T cells (considered recent CD4^+^ thymic emigrants) show inconsistent associations with physical activity (109, 127). Moreover, and importantly, observed associations between physical activity and CD8^+^CD45RA^+^CD103^+^ T cells (considered recent CD8^+^ thymic emigrants) are confounded by the use of CD103 to classify thymic emigrants (109). CD103 was thought to represent a robust T cell marker delineating new thymic emigrants (139), but recent research in rodents suggests that CD103 programming arises *after* thymic entry, specifically within the lymph nodes (140). Thus, elevated frequencies/proportions of CD103^+^CD8^+^ T cells in physically active people may not support the hypothesis that thymic output is augmented by physical activity. Interestingly, CD103 delineates a group of mature T cells that are preconditioned to migrate to peripheral tissues (140), where they commonly reside (141) and have an essential role in antigen – and tumour – specific responses (142). The potential importance of tissue-associated T cells in mediating the anti-cancer effects of physical activity is discussed in more detail later.

If physical activity is not augmenting thymic output to maintain the naïve T cell repertoire, then other factors that explain a high naïve T cell count should be considered. Indeed, in the absence of altered thymic output – which is deemed largely insufficient for maintenance of naïve T cell frequency in older age (143–145) – it may be the case that naïve T cells are instead maintained extra-thymically *via* homeostatic turnover (143) chiefly *via* cytokines including IL-7 (104). There is evidence from human studies that recombinant IL-7 administration can increase the T cell repertoire (146), and this process may culminate in enhanced eradication of previously non-immunogenic tumours (147). Therefore, given that physical activity – and specifically skeletal muscle – is implicated in elevated levels of homeostatic cytokines such as IL-7, it may be the case that physical activity does indeed maintain naïve T cells (109, 127), however more research is needed to confirm these observations. As described earlier, the role of IL-7 in maintaining naïve T cells is complex, and can result in naïve T cell differentiation resulting in the accumulation of memory T cells against self-antigens (105). Moreover, as discussed next, any relationship between homeostatic cytokines – and physical activity – with early differentiated T cells, is also likely to be highly dynamic.

#### Limitations of Immunophenotyping Approaches Used in Prior Studies

One important limitation shared among each of the aforementioned studies that assessed the relationship between physical activity or exercise training and ‘naïve’ T cell frequency/proportion in the blood, is the immunophenotyping approach used. As discussed above, most studies used one (e.g., CD45RA^+^ or CD28^+^) or two (e.g., CCR7^+^CD45RA^+^, or CD27^+^CD45RA^+^) cell surface markers to delineate ‘naïve’ T cells, but this introduces a key interpretational problem. Indeed, in the last decade it has been demonstrated that a long-lived population of memory cells exist which show profound multipotent capacity to proliferate, and are minimally differentiated unlike later differentiated memory T cells (e.g., central memory, effector memory T cells) (148). Importantly, these ‘stem cell-like memory’ T cells (T_SCM_ cells) share many features of naïve T cells including long telomeres and cell surface expression of CD45RA, CCR7, CD27, and CD28. Thus, any increase in so-called ‘naïve’ T cells reported in prior studies may not have been due to an increase in *bona fide* naïve T cells, but rather may be due to an increase in the maintenance of early differentiated memory T cells, with a phenotype akin to naïve T cells, such as T_SCM_ cells. If the latter is true, then physical activity may not be deleting memory T cells – as might be predicted here within Determinant 1 – but rather, physical activity may be *promoting* the maintenance of antigen-specific memory T cells. Moreover, early differentiated T cells – such as T_SCM_ cells – have been shown to mediate superior anti-tumour responses than their more differentiated antigen-specific memory T cell counterparts (e.g., central memory T cells and effector memory T cells, respectively) (148). This is likely due to their propensity to persistently differentiate. T_SCM_ cells have a profound ability to self-renew, in a process that is in part mediated by homeostatic cytokines, such as IL-7 and IL-15 (148, 149). Although the population of T_SCM_ cells in blood is thought to be small in healthy people (<5% of circulating T cells), this may contribute to explaining why there were associations found between homeostatic cytokines (e.g., IL-7, IL-15) and T cells with a naïve phenotype in prior studies (109, 127). Interestingly, it has been found that the T_SCM_: naïve T cell ratio increases during aging, and therefore the dynamic role of T_SCM_ cells in maintaining immunological memory may increase in ageing (150, 151).

A related consideration when seeking to interpret prior studies which have investigated the effects of physical activity on T cells, is the recent observation that T cells may not differentiate in a unidirectional linear pathway but rather demonstrate profound ‘plasticity’ that permits multiple cycles of phenotypic reversion (152), and the magnitude of ‘plasticity’ in T cell memory differentiation exceeds that predicted by more traditional views of a unidirectional T cell differentiation pathway (153). Indeed, it has been shown that memory T cells with a phenotype akin to naïve T cells can be generated from T cells exhibiting an effector memory T cell phenotype (152). These ‘naïve-revertant’ memory T cells (T_NRM_ cells) are capable of undergoing multiple cycles of phenotypic reversion (152), akin to T_SCM_ cells. Importantly, it has been shown that IL-7 and IL-15 stimulate the development of T_NRM_ cells following differentiation (152). Thus, it could be speculated that elevated homeostatic cytokines such as IL-7 and IL-15 in physically active people could revert – rather than delete – memory T cells, which may also explain the accumulation of naïve and early differentiated T cells in physically active elders (109), and after exercise training in older adults (128). Clearly, more research is needed to fully understand the effects of physical activity on the T cell compartment in blood.

#### Conclusion

In summary, a large body of research relating to Determinant 1 has focused on evaluating whether physical activity ameliorates the accumulation of senescent (e.g., CD57^+^) T cells and/or augments the maintenance of naïve T cells. However, there is a lack of robust evidence to indicate that physical activity reduces the accumulation of senescent T cells. It does however appear that physical activity promotes the accumulation of early differentiated T cells, including T cells with a naïve phenotype, in a process that may be governed by elevated homeostatic cytokines, such as IL-7, secreted from muscle. Whether or not these T cells are *bona fide* naïve T cells remains unresolved, and it may alternatively be the case that the higher frequency/proportion of T cells with a ‘naïve’ phenotype in physically active people is driven by better maintenance of early differentiated memory T cells, such as T_SCM_ cells or T_NRM_ cells. Later we outline how physical activity-induced immuno-regulatory changes to Determinant 1 might converge with other Determinants to explain how exercise regulates T cell function to maintain control of immunogenic cancer cells.

### Determinant 2 – Acute Exercise Alters T Cell Infiltration to Tissues Leading to the Enhanced Elimination of Immunogenic Cancer Cells

The second T cell-intrinsic determinant within the cancer immunogram is T cell infiltration to tumours (9). Research conducted in the field of exercise immunology/oncology relating to Determinant 2 has generally focused on one of the most reproducible findings in human physiology: exercise-induced lymphocytosis. Acute exercise induces a profound lymphocytosis by mobilising lymphocytes into circulation, *via* increases in cardiac output and shear forces flushing marginal venous pools (154) and *via* epinephrine-mediated detachment of lymphocytes from the endothelium (155). Within two to three minutes of exercise cessation, lymphocyte subpopulations in the circulation begin to decrease, and fall below resting levels within 30-60 minutes, known as lymphocytopenia (156). Late-differentiated effector cells with cytotoxic and tissue-migrating potential, e.g., CD56^dim^ NK cells and CD8^+^ T_EMRA_ cells, show the largest proportional increase following acute exercise, compared to less-differentiated naïve, central memory, and effector memory T cells, attributed to higher β2-adrenergic receptor expression on the surface of these cells (157, 158). Due to dose-response increases in epinephrine with increasing exercise intensity, the mobilisation of CD8^+^ T_EMRA_ cells is ~40-60% greater following high *vs.* low intensity exercise (157). It has been speculated that the mobilisation and egress of these highly cytotoxic immune cells in response to stress hormones may confer immune surveillance, for example in the identification and elimination of neoplastic cells residing in peripheral tissues as part of an evolved fight or flight response to stress hormones (159).

It is clear that the immune cells mobilised into the blood during exercise – and the same cells which are thought to egress after exercise – exhibit profound cytotoxic potential. Indeed, proportional increases in cytotoxic effector cells following acute exercise underpin increases in anti-cancer cytotoxicity *in vitro* in samples from humans. For example, NK cell cytotoxicity against nasopharyngeal carcinoma cells *in vitro* increases following an exhaustive ramp cycling test (160). Similarly, immediately after cycling for 30 minutes at 15% above lactate threshold, immune cell killing of five different haematological cancer cell lines was increased *in vitro* compared to killing at rest (161). Whilst it was noted that NK cells elicited much of the killing, it should be noted that CD8^+^ T cells likely contributed to cancer cell killing in this study (161), as CD8^+^ T cells are also profoundly mobilised by acute exercise (157) and because assays were performed with peripheral blood mononuclear cells containing T cells – which display cross-reactivity against non-self human leukocyte antigen (HLA) (162). For example, the increase in cancer cell killing from rest to immediately post-exercise was more pronounced for HLA^+^ cell lines (~110% increase) than HLA^−^ cell lines (~60% increase) (161).

#### Interpreting Findings From Animal Studies

Given the clear evidence that exercise mobilises T cells (and NK cells) with profound tumour cell killing potential, it is tempting to suggest that this acute mobilisation response underpins the anti-cancer effects of habitual physical activity (163). A range of rodent models have been used to investigate whether exercise-induced mobilisation of cytotoxic effector cells, and subsequent egress into tumours, underpins reductions in cancer growth. For example, mice that performed intensive treadmill running one day before injection of EL-4 lymphoid cells and exercised daily thereafter, displayed faster tumour rejection compared to non-exercising mice, due to increases in tumour-infiltrating lymphocytes which peaked at the onset of tumour regression (164). Furthermore, in a landmark study where voluntary wheel running was shown to suppress the development of injected and carcinogen-induced tumours by ~60% compared to non-exercising controls, homing of NK cells to tumours was identified as the mechanism mediating reductions in cancer growth (24). Importantly however, in one experiment, the greatest reductions in B16 tumour volume occurred in mice that ceased exercise before tumour challenge and did not continue with exercise after, whereas animals that only exercised after tumour challenge showed similar tumour volumes to non-exercise controls (24). As acute exercise-induced mobilisation of NK cells would have occurred in the days prior to the injection of B16 melanoma cells, the NK cells mobilised by exercise would not therefore have been able to infiltrate, recognise, and eliminate B16 tumour cells. Instead, the protective effects of exercise against cancer growth must have been an adaptive training effect, not resulting from transient acute effects following each exercise bout. Indeed, a recent study showed that the growth rate of B16 melanoma was suppressed in sedentary mice that received CD8^+^ T cells from exercise-trained mice, compared to sedentary mice transplanted with CD8^+^ T cells from untrained mice (165). Thus, it appears that alternative factors – beyond those arising in response to a single isolated bout of exercise – are important to the anti-cancer effects of exercise, and that NK cells might be less critical. Indeed, an often overlooked feature of the aforementioned study by Pedersen and colleagues (24) is that the greatest reductions to B16 tumour volume were observed in exercised mice with functional T cells (i.e., wild-type mice). It was shown – in Supplemental Figures 3C, D (24) – that B16 tumour mass was ~3 times smaller in exercised wild-type mice than non-exercise wild-type controls. In addition, and importantly, B16 tumour mass was ~2 times smaller in exercised wild-type mice than exercised mice lacking functional T cells (i.e., athymic mice); the latter exercised athymic mice exhibited control of very large cancerous lesions compared to non-exercising counterparts, but growth of smaller lesions was considerably less averted (24). Together, this suggests that exercise might augment NK cells against advanced-stage disease (i.e., B16 mutations that accelerate immune escape, such as changes to MHC-1 (166) but exercise-induced regulation of T cells is needed to maintain control of early-stage cancers. These important findings align with our observations from Part 2, where it was indicated that physical activity likely eliminates immunogenic cancer outgrowth in a process driven primarily by T cells. As stated earlier in Part 2, the majority of early-stage cancers express MHC-1 (48) and therefore maintenance of those cancers in the equilibrium phase of the immunoediting process is likely dictated by T cells rather than NK cells which tend not to be present in early-stage tumours (48, 49). On the other hand, exercise-induced modification of NK cells may play a role in helping to control late-stage disease (24).

When interpreting the animal studies within Determinant 2, it is important to consider that a range of different methods have been used to induce cancer in the rodents in these studies – including by transplantation, carcinogen administration, and transgenic approaches – and the degree of immunogenicity of the cancers induced by these approaches varies considerably. Importantly, it seems that the magnitude of tumour suppression attributed to acute-exercise mobilisation and infiltration of cytotoxic effector cells is diminished in transgenic models – the experimental model that most closely reflects the spontaneous and endogenous development of cancer in humans – when compared to injection of foreign cancer cells or carcinogen-induced models. For example, transgenic rodent models have shown that voluntary wheel running in Tg(Grm1)EPv mice tended only to *delay* the formation of melanoma lesions compared to non-exercising mice, but exercised mice were not spared the development of cancerous lesions; it is unclear if exercised animals exhibited differences in tumour volume or weight compared to non-exercised animals (24). In another rodent study, the number of colon polyps in transgenic Apc^Min/+^ mice allocated to treadmill running or non-exercise control was not different, but the number of large (>2 mm) polyps was reduced in exercising mice (72). Furthermore, regular voluntary wheel running did not suppress the development of mammary tumours in transgenic MMTV-PyMT mice, yet when a MMTV-PyMT mammary cell line (I3TC) was injected into wild-type rodents, voluntary wheel running (two weeks pre-injection, eight weeks post-injection) did suppress mammary tumour development (165). These important studies suggest that, in transgenic cancers containing cells of varying immunogenicity, exercise training does not always lead to the eradication of cancer, and rather, only the most immunogenic cancer cells are eliminated. Instead, a delayed outgrowth or equilibrium of more benign tumours is achieved (24, 39, 72). Thus, using other methods such as injecting highly immunogenic foreign cancer cells (e.g., tumour cells lacking MHC-1) into rodents may exaggerate the anti-tumour effects of exercise when compared with transgenic models. Additionally, these models also exaggerate the importance of NK cells as the underlying mechanism-of-action of exercise. Even when foreign cancer cells are injected into rodents, it appears that T cells may play a more profound role in the exercise response than NK cells (165). This mechanistic deduction aligns with human epidemiology studies, as well as rodent studies assessing spontaneous cancer development, which suggest that physical activity tends to delay outgrowth by maintaining cancers in the equilibrium phase of immunoediting – and central to the maintenance of equilibrium is the adaptive immune system, and specifically T cells (42).

#### Does an Acute Bout of Exercise Preferentially Mobilise Immune Cells to Tissues?

Whilst it seems logical that the anti-cancer effects of physical activity are predominantly derived from T cells, it is important to consider whether this response is reliant on the acute mobilisation (into blood) and egress (into tissues) of T cells in response to individual bouts of exercise. An important study in rodents which assessed the effects of propranolol – a beta blocker which nullifies immune cell mobilisation and egress during acute exercise – on immune cell migration to tumour sites, showed – in Supplemental Figure 4C of the article by Pedersen and colleagues (24) – that T cells were substantially affected by propranolol blockade and this coincided with abrogation of tumour suppression (24). This may indicate that CD8^+^ effector T cells – which express the greatest surface density of β2-adrenergic receptors, compared to other T cell subsets (167) – may be trafficking from blood in response to exercise, and the trafficking of these cells appears to be abrogated by propranolol (24). Nevertheless, it remains unclear whether acute exercise is driving the migration. For example, it is thought that beta-adrenergic stimulation regulates effector T cell migration patterns, at rest, in the absence of exercise (168). Indeed, the magnitude of blood effector CD8^+^ T cell frequency at rest – but not other less-differentiated CD8^+^ T cell subsets – is determined strongly by epinephrine and fluctuates profoundly throughout the day, and this process is thought to facilitate homeostatic effector T cell migratory potential (168). In the absence of sufficient beta-adrenergic stimulation, effector memory T cells commonly attach to endothelial cells in the so-called marginal pools (169). Therefore, blockade of diurnal migratory processes, *via* propranolol, may therefore inhibit diurnal effector T cell migratory potential, independently of any migratory effect induced from acute exercise. A separate limitation of the acute-exercise hypothesis is that it assumes that upon any acute mobilisation to tissues that the tumour – at a snapshot in time – would be overtly immunogenic, but as the cancer immunogram dictates, this is not a certainty. Nevertheless, it seems that immune cell migration – and specifically migration of T cells – appears to be important in the anti-cancer immune response elicited by exercise, regardless of whether the infiltration of T cells arises in response to acute exercise or not (24). However, it could be that the increased accumulation of effector T cells in tissue harbouring tumours could be driven by a separate factor that arises as a result of exposure to regular exercise, discussed next.

The relevance of the acute mobilisation response (in blood) and subsequent trafficking to tumour sites (in peripheral tissues and organs) is further clouded by a lack of evidence to show that a single bout of exercise causes immune cells to migrate to peripheral tissues and organs. Animal models using fluorescent tracking suggest that T cells may egress from the circulation to the lungs, Peyer’s patches, and bone marrow to conduct immunosurveillance following acute exercise (158). However, the reported egress of T cells into tissues harvested immediately post-exercise may instead be a confounding effect of enhanced blood flow to the lungs (170) and bone marrow (171) during acute exercise, and to the digestive tract during exercise performed in a post-prandial state (172). It is therefore important to consider how physical activity may affect immune cells – and specifically T cells – in the tissues, in a manner that is not reliant on individual bouts of exercise.

In doing this, it is important to consider that it is estimated that only ~2% of all lymphocytes are found in the circulation in humans (173) and the majority are located within peripheral tissues and organs. Tissue-associated T cells – also dubbed ‘tissue-resident’ memory T cells (T_RM_ cells) – commonly expressing the cell surface markers CD69^+^ or CD69^+^CD103^+^ are abundantly located within the skin, respiratory tract, brain, heart, dorsal root ganglia, intestines, vaginal mucosa, salivary glands, bone marrow, thymus, lymph nodes, spleen, brain, liver, kidneys, pancreas, and white adipose tissue, and undergo limited or no recirculation in blood (174, 175). In addition, innate immune cells such as NK cells and type 1 innate lymphoid cells occupy the lungs, lymph nodes, liver, uterus, and skin (176, 177) and the small intestine, lamina propria, salivary glands, lungs, and liver (178), respectively. The presence of NK cells in these tissues questions the need for additional NK cells to be present, given that innate immune cells unilaterally elicit cytotoxicity against cancer cells with reduced MHC-1. The anti-cancer properties of these tissue-resident effector cells have received recent research attention in both human and rodent studies (179). Studies in humans with a clinical cancer diagnosis have shown that prognosis is more strongly associated with tumour-infiltrating CD8^+^ T cells expressing tissue-residency marker CD103^+^ than total tumour-infiltrating CD8^+^ T cells (180, 181). Furthermore, rodent studies have implicated T_RM_ cells in anti-tumour immunity, by showing that mice lacking CD69 or CD103 or other T_RM_-associated molecules (e.g., CD49a) are more susceptible to transplanted B16 melanoma (182–184). As such, it may be that acutely trafficking additional circulating effector cells to tissue sites abundantly occupied by tissue-resident effector cells may not underpin reductions in tumour volume seen in studies comparing exercising and non-exercising rodents, and perhaps these tissue-associated cells are instead supplemented in another manner by physical activity.

Indeed, if acute exercise-induced ingress of blood T cells does not underpin the anti-cancer effects of exercise, then exercise must instead modulate tissue-associated T cells in another way. As outlined in Determinant 1, the maintenance of blood T cell phenotypes is strongly influenced by homeostatic cytokines, including IL-7 and IL-15. Interestingly, these same immunoregulatory cytokines (IL-7 and IL-15) are thought to be implicated in the development and maintenance of tissue-residing T_RM_ cells as well as other type 1 innate lymphoid cells (185, 186), which have the capacity to exert cytotoxicity against neoplastic cells (184, 185). As highlighted in Determinant 1, physically active individuals exhibit higher blood levels of IL-7 and IL-15 (109, 127), which may in turn be associated with the regulation of early differentiated memory T cells (e.g., T_SCM_, T_NRM_) in blood. These T_SCM_ and T_NRM_ cells also have profound and rapid multipotent differentiation capacity and likely act as a memory stem cell for T_RM_ cells in the tissues (187). Therefore, it may be the case that the augmentation of circulating immunoregulatory cytokines in physically active people influences the maintenance of memory T cells in both blood and the tissues. And, in the event of T_RM_ cell activation in the tissues, these blood T_SCM_ or T_NRM_ cells rapidly differentiate and infiltrate the tissues to assist T_RM_ cells in the elimination of immunogenic clones. Such an example removes the need for acute-exercise induced-mobilisation of immune cells to fulfil Determinant 2. Nevertheless, there is still a lack of clarity about how acute and regular exercise mediates changes to T cells in the tissues, and more research is needed to discern how exercise alters T cell immunosurveillance.

#### Conclusion

Overall, the animal studies discussed in Determinant 2 support the theory that exercise delays the outgrowth of cancer, as a result of enhanced elimination of immunogenic cancer cells by the immune system – thus mirroring the effects of physical activity seen in human epidemiology studies, as outlined in Part 1 of this review. There is some evidence that the anti-cancer effects of physical activity are mediated by the acute mobilisation and redistribution of cytotoxic effector cells in response to single bouts of exercise, but a number of limitations to this theory were identified. Rather it seems that the anti-cancer immune effects of physical activity may be principally driven by an adaptive training effect that augments tissue-associated T cells. Central to this effect appears to be the modulation of tissue-associated T cells in a process that is independent of the acute exercise-induced lymphocytosis response. As an example, it may be that tissue-associated T cells are regulated directly by muscle-derived homeostatic cytokines such as IL-7 and following activation in the tissues these may be further nourished by early differentiated memory T cells (e.g., T_SCM_), which are maintained in the circulation by homeostatic cytokines including IL-7. Later we outline how aspects of Determinant 2 might converge with other Determinants to explain how physical activity augments immune competency to promote the elimination of immunogenic cancer cells.

### Determinant 3 – Physical Activity Alters Immune Checkpoints Associated With T Cell Anergy and Exhaustion in the Tumour Microenvironment

As described in Determinant 1 and 2, it is clear that the regulation of memory T cells in blood and at sites of cancer development may be important in contributing to the anti-cancer effects of physical activity. However, the tissue microenvironment in cancer induces anergy and exhaustion to effector cells residing in tumours, due to immunosuppressive signalling and chronic antigen stimulation, as described by the ‘immune checkpoints’ component of the cancer immunogram (9). Central to Determinant 3 is the theory that physical activity may alleviate features of anergy and exhaustion, leading to a greater likelihood of immunogenic cancer cell elimination in physically active individuals. Indeed, 9/13 cancers shown to exhibit reduced incidence in individuals with the highest physical activity level (2) – head and neck, liver, kidney, lung, breast, gastric cardia, colon, rectum, and bladder cancers – are also responsive to treatment with immune checkpoint inhibitors that alleviate T cell anergy by inhibiting cytotoxic T lymphocyte-associated protein (CTLA)-4 or T cell exhaustion by inhibiting programmed cell death protein (PD1) and/or its ligand (PDL1) (188). Together, the close agreement between cancers responsive to immune checkpoint inhibitors and physical activity implies the involvement of a similar anti-cancer mechanism-of-action *via* T cells, which may arise *via* the resolution of T cell anergy and exhaustion. In this section, we begin by summarising how T cell anergy compromises the elimination of cancer cells, followed by studies demonstrating that physical activity or exercise training may alleviate T cell anergy. Subsequently, the role of T cell exhaustion in immune escape is discussed, followed by preliminary evidence examining associations between physical activity and reductions to features of T cell exhaustion.

#### T Cell Anergy

Anergy describes T cells in a hyporesponsive state due to suboptimal co-stimulatory signalling *via* CD28, and/or high co-inhibitory stimulation, which is a functional mechanism of peripheral tolerance to protect the host from autoimmunity (189). T regulatory (T_REG_) cells maintain peripheral tolerance *via* immunosuppressive cytokines IL-10, transforming growth factor (TGF)-β, and IL-35, suppressing costimulatory CD80/CD86 receptor expression on antigen-presenting cells, and *via* CTLA4 binding to CD80/CD86 with higher affinity than CD28 expressed by effector cells (190). Increases in T_REG_ cell frequency – independent of detriments to effector T cells – have been shown to underpin reductions in immunity to infectious disease in older adults (191). As the risk of clinically diagnosed cancer increases with advancing age (38), increased T_REG_ cell frequency in older adults may also underpin detriments to anti-cancer immunity that result in a clinical cancer diagnosis when preclinical cancer escapes immune control. Further evidence in support of the role of T_REG_ cells in suppressing anti-tumour immunity is the efficacy of anti-CTLA4 immunotherapy in the treatment of certain cancers (188) which primarily acts to restore effector T cell activation and function *via* the depletion of T_REG_ cells expressing CTLA4^+^ (192, 193). As such, modulation of T_REG_ cell frequency *via* physical activity may represent a mechanism by which anergy is resolved and anti-cancer immune effector function is unleashed to eliminate immunogenic cancer cells.

#### T Cell Anergy and Physical Activity

Effector CD4^+^ T cells and CD4^+^ T_REG_ cells are generated concurrently during memory immune responses (194) and the differentiation of naïve CD4^+^ T cells to T_REG_ cells or Th1 effector cells is determined by the cytokine milieu (195). Therefore, given that physical activity is associated with altered blood concentrations of immunoregulatory cytokines, it may be the case that physical activity influences T_REG_ cell development. Indeed, IL-15 – which is elevated in individuals with a higher physical activity level (109, 127) – preferentially supports the proliferation, effector function, and survival of effector T cells compared to T_REG_ cells (196) and has been shown to blunt the suppressive effects of T_REG_ cells on effector T cells (197). Furthermore, in the context of cancer immunotherapy, IL-15 has been shown to restore the anti-tumour cytotoxicity of anergic CD8^+^ T cells against injected erythroleukaemia cell lines in mice *in vivo* (198) and to restore interferon (IFN)-γ production by Wilms’ tumour antigen-specific T cells *ex vivo* in patients with Wilms’ tumour antigen-expressing prostate cancer (199). Meanwhile, elevated circulating concentrations of IL-7 in physically active individuals (109, 127) may also be beneficial as IL-7 does not support the survival and function of T_REG_ cells, due to downregulation of IL-7 receptor (CD127) on T_REG_ cells (200, 201) whereas it favours the maintenance of memory T cells, thus enhancing immune effector capabilities to maintain cancer in equilibrium (8, 41, 42).

While the mechanisms of T_REG_ downregulation and resolution of T cell anergy in physically active individuals require confirmation, cross-sectional studies in humans have found lower circulating proportions of T_REG_ cells (CD4^+^CD25^+^FoxP3^+^) in individuals self-reporting a higher physical activity level (109, 202). Yet, cross-sectional studies utilising device-based measurements of physical activity showed no difference in the proportion of T_REG_ cells (CD4^+^CD25^+^CD127^Lo^ or CD4^+^CD25^+^FoxP3^+^) between individuals performing ~1000 kcal/day *vs.* ~500 kcal/day of active energy expenditure (203) and older adults performing >10,500 *vs.* <5,000 steps/day (127). None of the aforementioned studies measured CTLA4^+^ expression on T_REG_ cells. Intervention studies also show inconsistent effects of exercise training on T_REG_ cells, which may be explained by differences in phenotyping approach, exercise prescription, or patient population. For example, CD4^+^CD25^+^CD127^−^FoxP3^+^ T_REG_ cells have been shown to decrease following 20 weeks of exercise training in individuals with rheumatoid arthritis (204). In contrast, T_REG_ cells identified *via* CD25^+^ expression on CD4^+^ T cells increased following 12 weeks of tai chi training (205). Furthermore, tai chi training increased FoxP3 mRNA expression in total leukocytes of individuals with type 2 diabetes, but not in age-matched individuals without diabetes (206).

Despite mixed effects of exercise training on blood T_REG_ cells in humans, the beneficial effects of reducing T_REG_ cells, and other immunosuppressive cell populations, *via* exercise training has been shown in rodent models of cancer. Indeed, in preclinical rodent studies where the growth of experimentally-induced tumours was suppressed by exercise, reductions in immunosuppressive cell populations, or expression of chemokines involved in recruiting suppressive immune cells, within tumours underpinned reductions in tumour outgrowth. For example, in a transgenic colon carcinogenesis model, a 48% reduction in the development of large polyps in Apc^Min/+^ mice after 12 weeks of treadmill running occurred alongside reductions in FoxP3^+^ expression, and expression of M2 macrophage genes – including T_REG_ recruitment chemokine CCL22 – in colon mucosal tissue (72). In another study, the size of mammary tumours in transgenic PyMT mice was reduced in exercising *vs.* non-exercising mice during weeks 1-3 of voluntary wheel running, associated with reductions in per-macrophage CCL22 expression, which may have reduced T_REG_ recruitment (207). Furthermore, protective effects of exercise were lost from weeks 3-10, suggesting exercise only suppressed the early stages of cancer development, i.e., the equilibrium phase (207).

Reductions to tumour-infiltrating immunosuppressive cells have also been shown to help repress the outgrowth of injected cancer cells in exercising mice. For example, exercise performed for eight weeks prior to subcutaneous 4T1 injection reduced tumour growth rate by 17%, alongside reductions in FoxP3^+^ T_REG_ cells within tumours compared to non-exercising rodents (208). Further, when exercise was performed regularly after subcutaneous 4T1 injection, tumour growth rate was slowed alongside reduced expression of immune-suppressive markers GATA3, RORyT and IL-10 in tumour-infiltrating CD4^+^ cells (209) and proportional reductions to myeloid-derived suppressor cells within tumours (210). In addition, regular exercise performed before and after subcutaneous injection with Hepa 1-6 liver cancer cells reduced tumour infiltrating CD25^+^FoxP3^+^ T_REG_ cell proportion, alongside a 19% reduction in tumour growth (211). Overall, exercise training performed before and/or after tumour induction – *via* genetic alterations or cancer cell injection – in rodents appears to reduce the accumulation of regulatory immune cells within tumours to suppress cancer development.

#### T Cell Exhaustion

Exhausted T cells develop in the presence of chronic antigen stimulation and can be identified by the expression of inhibitory receptors (e.g., PD1) which prevent T cell activation (189). Indeed, expression of inhibitory receptors has been shown to inhibit downstream signalling pathways that regulate T cell proliferation and survival, resulting in a loss of IL-2, tumour necrosis factor (TNF)-α and IFN-γ production (212). Functional T cell exhaustion develops in chronic viral infections and autoimmune conditions, where it is considered an adaptive mechanism to limit tissue damage and dampen autoimmune reactions, but may also compromise anti-cancer immunity (212). For example, idiopathic pulmonary fibrosis is associated with increased expression of PD1^+^ on peripheral blood CD4^+^ T cells and within lung tissue (213) and individuals with pulmonary fibrosis are at increased risk of developing lung cancer (214). The presence of functionally exhausted T cells within these tissues – which are compromised in their ability to eliminate cancer cells – may explain the increased risk of a clinical cancer diagnosis within affected tissues. Furthermore, when preclinical cancer develops, effector T cells continuously encounter neoantigens, along with immunosuppressive cytokines e.g., IL-10 and TGF-β, which induces T cell exhaustion in the tumour microenvironment (215) representing a mechanism by which cancer escapes immune control and grows aggressively (216). Studies have identified high circulating proportions of CD8^+^PD1^+^ T cells as an adverse prognostic factor in cancer (217, 218). Furthermore, an elevated PD1/CD8 ratio within tumour-infiltrating lymphocytes is associated with worse cancer prognosis (219, 220). As such, inhibitory receptors are a target of cancer immunotherapy. Indeed, the efficacy of anti-PD1 immune checkpoint inhibitors in certain cancers demonstrates the potential of ameliorating T cell exhaustion to reinvigorate the host anti-cancer immune response and eliminate cancer cells (221).

#### T Cell Exhaustion and Physical Activity

The role of exercise in immunotherapy – including immune checkpoint blockade – has recently been reviewed elsewhere (222). Briefly, to date, only one study in humans has shown that more physically active individuals display a lower proportion of PD1^+^ CD4^+^ T cells - and possibly PD1^+^ CD8^+^ T cells - compared to individuals with a lower physical activity level. A series of preclinical rodent studies have explored the effects of exercise training on T cell exhaustion and cancer growth, however results are mixed. One study showed that exercise training following 4T1 breast tumour injection in combination with radiotherapy and anti-PD1 immunotherapy reduced the proportion of splenic PD1^+^CD8^+^ T cells – where anti-cancer therapy alone did not – and reduced tumour growth (210). The effect of exercise alone on PD1^+^CD8^+^ T cells was not reported, however, the addition of exercise to radiotherapy and immunotherapy appeared to potentiate the effectiveness of anti-cancer therapy (210). In another study, where regular exercise was performed prior to, and two weeks after, subcutaneous injection of B16 melanoma cells, PD1 expression in tumours was higher in exercising *vs.* non-exercising rodents, yet tumour growth was suppressed in exercising rodents, suggesting the involvement of other mechanisms in mediating the anti-cancer effects of exercise (223). Furthermore, in contrast to the aforementioned findings (210) there were no additive effects of exercise and anti-PD1 immunotherapy (223). Finally, in a third study, exercise training performed before and after tumour injection had no effect on the expression of PD1 on non-small cell lung cancer tumour tissue, and tumours grew most quickly in mice treated with combined anti-PD1 immunotherapy and exercise, suggesting detrimental effects of combination therapy (224); importantly, the mouse model utilised in this research was devoid of lymphocytes and thus reliant on anti-tumour effects elicited by neutrophils, monocytes, and other factors (224). Taken together, these collective findings indicate that the effects of exercise training on both (i) PD1^+^ T cells and (ii) the efficacy of anti-PD1 immunotherapy remain inconclusive. Further research is required to understand whether the anti-cancer effects of physical activity are mediated by pathways dependent or independent of PD1.

In considering this conundrum, it may be important to consider that the restorative effects of checkpoint inhibitor therapies appear to be mediated by a distinct ‘stem cell-like’ subset of CD8^+^ T cells, which proliferate to a greater extent following anti-PD1 therapy than terminally-differentiated exhausted CD8^+^ T cells (225, 226). Stem cell-like ‘exhausted’ CD8^+^ T cells are distinguished from their terminally-differentiated counterparts *via* expression of T cell factor-1, CD28 and CD127 (227). Indeed, stem cell-like PD1^+^CD8^+^ T cells express CD127 – the IL-7 receptor – and this is downregulated on CD8^+^ T cells subjected to persistent antigen stimulation (228). The restorative effects of IL-7 on T cell exhaustion have been demonstrated in a range of rodent studies (229, 230). Notably, IL-15 also appears to exhibit similar effects (231), and may additionally blunt the suppressive effects of T_REG_ cells (197) which produce TGF-β and IL-10 that induce T cell exhaustion (215). As previously outlined, in Determinant 1 and Determinant 2, immunoregulatory cytokines, such as IL-7 and IL-15, circulate at higher concentrations in physically active persons (109, 127) and induce alterations to T cell plasticity and function. Therefore, there may be indirect mechanisms through which physical activity can augment anti-tumour effector function in a manner that is synergistic with PD1 immune checkpoint therapy. Moreover, as discussed later, these effects appear to be intertwined with the effects of physical activity on inflammatory (Determinant 4) and metabolic (Determinant 5) suppressors of T cell function within the tumour microenvironment. Thus, physical activity may prove to be a powerful adjunct to anti-cancer therapy by altering many aspects of the cancer immunogram (9), which may support T cell effector function against immunogenic cancer cells.

#### Conclusion

In summary, physical activity may resolve effector cell dysfunction in the tumour microenvironment to suppress cancer development. Indeed, reductions to T cell anergy and exhaustion may contribute to the enhanced elimination of cancer cells to maintain subclinical equilibrium in physically active individuals. Later, we outline how the restorative effects of physical activity on the function of anergic and exhausted T cells may combine with immunologic adaptations outlined in other Determinants, which may explain how physical activity augments the elimination of immunogenic cancer cells.

### Determinant 4 – Physical Activity Alters the Presence of Inflammatory Inhibitors of T Cells in the Tumour Microenvironment

The fourth T cell-intrinsic determinant within the cancer immunogram is T cell inhibition by pro-inflammatory mediators in the tumour microenvironment (9). This determinant of T cell function predicts that the presence of pro-inflammatory mediators, including IL-6, CRP, and other factors, inhibits T cell effector function and culminates in poor clinical outcomes in many cancers (9).

To date, research assessing the effects of physical activity on inflammatory inhibitors has largely centred on systemic inflammation in blood, with fewer studies assessing exercise-induced changes to inflammation in local tissues and the tumour microenvironment. It is beyond the scope of this review to summarise the extensive literature investigating the relationship between physical activity and inflammatory mediators, and here we briefly discuss how physical activity affects IL-6 and CRP, as these are among the most studied inflammatory biomolecules in exercise science, and both are highlighted within the cancer immunogram as biomarker examples of an inhibitory T cell environment.

Briefly, it is widely reported that a higher physical activity level is associated with lower IL-6, CRP, and other pro-inflammatory mediators in blood (232). Whilst cross-sectional studies in humans largely support this contention, the findings from randomised-controlled exercise trials are mixed (233). Some studies conducted in middle/older age adults demonstrate that regular aerobic exercise training reduces IL-6 and CRP (234, 235), whereas others found no effect on these biomarkers (236). Nevertheless, a recent systematic review and meta-analysis identified that exercise training does result in reductions to both IL-6 and CRP, as well other inflammatory mediators (237). Given these observations, it is important to consider the stimulus for elevated IL-6, CRP – and other markers of inflammation – in people who are less physically active. It is thought that heightened levels of IL-6, CRP, and other pro-inflammatory mediators in less physically active people are largely linked to higher adipose tissue, which in turn contributes to a pro-inflammatory phenotype (238). Moreover, there is evidence that reductions to systemic IL-6 and CRP from exercise training are reliant on body mass-loss (239). Importantly, the association between physical activity and reduced clinical cancer risk appears to be independent of body mass (1, 2), which questions the role of systemic inflammation – associated with adiposity – in the anti-cancer effects of physical activity (240). Alternatively, higher levels of systemic inflammation are thought to be associated with physical inactivity, in a manner independent of adiposity (238, 240). This may be due to age-associated inflammatory dysregulation, also known as ‘inflammageing’. Inflammageing is in part thought to represent the chronic activation of immune effector cells (due to subclinical disease/damage) and other factors including sarcopenia, and coincides with increased levels of pro-inflammatory mediators, including IL-6 and CRP, in blood (241), which in turn is associated with increased risk of morbidity and mortality in older age (242). Thus, physical activity may alter inflammatory mediators in a manner that is independent of adiposity. Regardless of the systemic stimulus of IL-6, CRP (and other inflammatory mediators), it might be the case that physical activity is sufficiently capable of altering the local inflammatory milieu to favour T cell reactivity. Nevertheless, this is likely to be an over-simplification because, as discussed next, the effects of physical activity on inflammatory mediators in the tumour microenvironment is complex.

A growing number of studies have explored the impact of exercise on inflammatory mediators in the tumour microenvironment. Using IL-6 as an example, in an aforementioned study assessing the effects of regular exercise on liver disease and cancer in nfkb1^-/-^ mice, it was reported that exercise resulted in lower levels of IL-6 and other signs of inflammation in the liver, as well as other tissues, and this was linked to reduced cancer development (73). Other studies have shown that – rather than decrease the availability of local IL-6 – exercise in rodents resulted in an increase to IL-6 gene expression within B16 melanoma tumours following voluntary wheel running, and this was accompanied by a 60% reduction to tumour growth (24). Similarly, where mammary tumour growth was suppressed by 52% in mice that were subjected to stretching exercise compared to non-stretching mice, IL-6 was upregulated within tumours (243). Moreover, the availability of IL-6 is thought to act as a stimulus for immune effector cell infiltration to tumours in response to regular exercise (24). Indeed, in the same study, tumour suppression was abolished upon treatment with anti-IL-6 monoclonal antibodies (24); the inhibition of immune effector responses, which in part is co-ordinated by IL-6, may explain this finding. Indeed, IL-6 signalling is pleiotropic and highly complex, and alongside its well-known tumour-promoting effects, it paradoxically promotes effector T cell priming within lymph nodes and subsequent trafficking to tumour sites, which in turn suppresses tumour development (244). Clearly therefore, it might be too simple an expectation to solely rely on an exercise-induced reduction to IL-6 (and CRP) to promote a T cell reactive environment in the tumour.

In considering this problem, it is important to contemplate that the presence of IL-6 and other related inflammatory inhibitors of T cells in the tumour microenvironment is likely a bi-product of a range of different cell types, including tumour cells, stromal cells, and immune cells. For example, cancer cells secrete tumour-promoting cytokines such as IL-6, TNF-α, and IL-1 which – along with potentiating their own growth and survival – leads to the recruitment of immunosuppressive immune cells to the tumour microenvironment (245). Upon recruitment and activation of tumour-associated macrophages and myeloid-derived suppressor cells, these immune cells release IL-10 and TGF-β, and this concomitantly results in the recruitment of T_REG_ cells which themselves produce IL-10 and TGF-β (246). As discussed earlier in Determinant 3, the aforementioned immunosuppressive cytokines induce anergy (190) and exhaustion (215) to effector T cells, which hampers the elimination of immunogenic cancer cells and facilitates tumour escape from immune control. Given that immune cells are a major source of inflammation in the tumour, it should be considered that physical activity also has an impact on immune cell kinetics in peripheral tissues and within tumours – as outlined in the other Determinants herein. Therefore, in assessing the effects of physical activity on tumour inflammation, it should also be considered how exercise impacts immune cells, as this likely contributes to changes to the inflammatory phenotype within the tissue being investigated.

A final consideration which is important to consider, is that – as outlined in Part 2 – the resolution of inflammatory dysregulation is also commonly cited as an anti-cancer mechanism of physical activity which *directly* regulates tumour growth in the absence of immune involvement by withdrawing proliferative stimuli to reduce the risk of initiating mutations and the promotion of cancer (40). On one hand, a wealth of evidence supports a role for cytokines, such as IL-6, in promoting tumour progression by increasing proliferation, survival, and invasion of cancer cells (245), thus it could be deduced that reducing IL-6 through physical activity may help to avert cancer. However, it is notable that blood IL-6 is weakly associated with pan-cancer risk (247). Furthermore, as an acute bout of exercise results in a rapid ~100-fold increase in blood IL-6 (248) it might be expected that individuals performing regular exercise – and thus regularly exposed to high amounts of IL-6 – would have increased risk of cancer, yet this is not the case (1, 2). Indeed, it is likely to be an over-simplification to expect that an isolated reduction to IL-6 corresponds with a direct reduction to cancer growth. As an example, breast cancer cells incubated with serum collected immediately after acute exercise – which contained 2.1-fold higher IL-6 than resting serum samples – displayed lower viability *in vitro* than cancer cells incubated with resting serum (249). Moreover, in the aforementioned study, it was shown that six months of exercise training reduced basal IL-6 in serum by 37%, yet this reduction to IL-6 did not reduce the viability of breast cancer cells *in vitro* (249). Thus, it is evident that the role of IL-6 and other inflammatory mediators in cancer progression is profoundly complex, and it is likely too simplistic to suggest that the withdrawal of IL-6 signalling from cancer cells *via* exercise training is sufficient to stifle cancer growth.

#### Conclusion

In summary, there is a plethora of evidence that physical activity reduces systemic inflammatory mediators such as IL-6 and CRP. Nevertheless, there is mixed evidence that regular exercise reduces these inflammatory mediators in the tumour microenvironment – in fact some studies show that IL-6 may be increased by exercise, and this corresponds to enhanced anti-tumour responses. It is likely that the heterogeneity of research findings to date reflects the fact that inflammatory cytokines are produced from a range of cell types, including tumour cells and immune cells, which are each affected by exercise in numerous ways – as outlined in the other Determinants within this review. Later, we outline how exercise-induced changes to the inflammatory milieu may combine with other immunologic adaptations to explain how physical activity augments the elimination of cancer cells.

### Determinant 5 – Physical Activity Alters the Presence of Metabolic Inhibitors of T Cells in the Tumour Microenvironment

The fifth T cell-intrinsic determinant within the cancer immunogram is inhibition of T cell function by metabolic features within the tumour microenvironment (9). This determinant of T cell success predicts that T cell effector function is inhibited by factors including intratumoural hypoxia, acidity, increased lactate, glucose depletion and other metabolic factors (9). It has been hypothesised that regular exercise may alleviate each of these inhibitory metabolic conditions – including hypoxia, acidosis, lactate accumulation, and reduced glucose – within the tumour microenvironment, which in turn reduces immune cell inhibition against tumour cells (250).

It is beyond the scope of this review to address how exercise affects each factor contributing to T cell-inhibitory metabolic dysregulation, and the reader is directed to a comprehensive review on this topic (250). Briefly, improvements to the metabolic profile of the tumour with exercise – including reduced intratumoural hypoxia, increased glucose availability and decreased acidity and lactate accumulation – is thought to predominantly arise as a result of increased blood flow (250). Indeed, acute exercise has been shown to increase tumour blood flow by up to 200% compared to rest (251, 252). Although, importantly, this finding does not extend across anatomical locations of tumours, with some sites experiencing exercise-induced reductions to blood flow when compared to resting conditions (252). Indeed, blood volume is directed to metabolically-active tissues, e.g., skeletal muscle, during acute exercise (253), and therefore, it may be the case that tumour perfusion is transiently suppressed during exercise for cancers which reside in non-exercising tissues.

Nevertheless, there is evidence that in the event physical activity augments tissue vascularisation and increases blood flow, this may lead to a more favourable metabolic profile in the tumour, including the alleviation of hypoxia (250). Tumour hypoxia is the result of oxygen demand exceeding supply in the context of highly-proliferative cells and disorganised tumour vasculature. For example, in rodent models of cancer, it has been shown that structured exercise training (e.g., treadmill running) increased tumour vascularity and perfusion, and reduced the size of hypoxic regions within tumours, compared to rodents that did not perform exercise training (254–258). However, it has also been shown that exercising rodents (ten days of treadmill running for 60 minutes/day) exhibited reduced tumour vascularity compared to non-exercising rodents (259). Additionally, whilst the tumour vascularisation theory of regular exercise may hold promise for potentially explaining some of the anti-cancer effects of physical activity against ‘solid’ tumours, it does not explain why physical activity reduces the incidence of many blood cancers (1, 2), which commonly recirculate between blood and lymphoid tissues.

Bringing consonance to the aforementioned problem, it may be that solid tumour vascularisation permits greater effector immune cell infiltration to solid tumours. Evidence linking exercise, tumour vascularity, and T cell function has emerged from a recent study, which showed that exercise training in rodents increased tumour vascularity, reduced hypoxia, and increased tumour infiltration of CD8^+^ T cells which exhibited enhanced effector function (260). Importantly, CD8^+^ T cell depletion abrogated the anti-tumour effects of exercise, thus demonstrating the central importance of CD8^+^ T cells in the anti-cancer response (260). In addition to CD8^+^ T cell infiltration, it is also possible that tumour vascularity can help rescue an immunosuppressive environment that is characteristic of hypoxic tumours – such as T cell anergy and exhaustion (261) and reduced MHC-1 expression (262) – but the effects of exercise on these outcomes needs investigating in future studies. However, it is notable that T cell-suppressive metabolic features – such as lactate accumulation and acidosis – may persist despite improved vascularity and oxygen delivery due to the so-called Warburg effect whereby tumour cells metabolise glucose to lactate, even in the presence of adequate oxygen (263).

It is also important to consider that physical activity has other effects on T cells – as outlined in Determinants 1-4 – which may alter the landscape of T cell infiltration and function within tumours, in a manner that is independent of those outlined here in Determinant 5. In such a scenario, if the composition of functionally competent effectors cells was favourably altered within the tumour by a factor other than vascularisation, then an accelerated eradication of immunogenic tumour cells – which consume oxygen and glucose and produce lactate – would still be expected, and in turn this would give rise to a tumour microenvironment that appears less metabolically restrictive to effector T cells. This highlights that when evaluating the role of physical activity on inhibitory metabolism in the tumour microenvironment at a snapshot in time, it is important to consider how physical activity has affected immunological profiles within the tumour microenvironment, as outlined in Determinants 1-4.

#### Conclusion

There is increasing evidence that regular exercise can improve tumour vascularisation, leading to greater effector T cell infiltration, and an altered metabolic profile that may be optimal for effector T cells, including reduced hypoxia. There is some limited evidence that these changes correspond to improved T cell function, but it is unclear if these metabolic effects of exercise are mediated by optimised T cell function, as highlighted in Determinants 1-4. Next, we briefly summarise how exercise-induced changes to the metabolic milieu may interact with other immunologic adaptations outlined in Determinants 1-4, to explain how physical activity might facilitate the maintenance of cancers within the equilibrium phase of the immunoediting process.

### Part 3 Synopsis – A Synthesis of Key Elements Highlighted in Determinants 1-5

**Figure 2:**
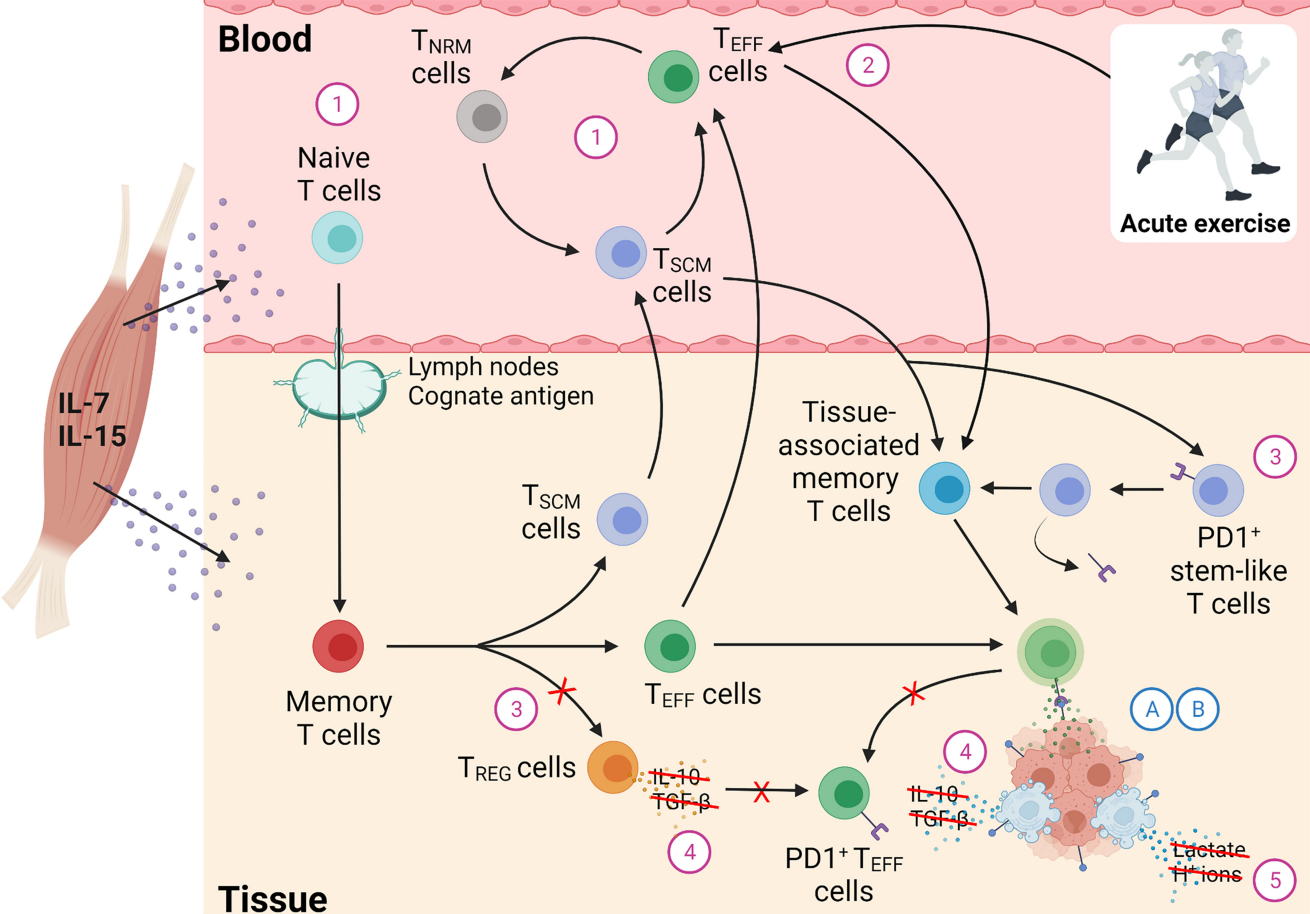
A synthesis of possible candidate mechanisms explaining how T cell regulation is augmented by physical activity leading to enhanced T cell function against tumour cells, culminating in the preservation of cancers in the equilibrium phase of the immunoediting process. In this hypothetical process, we illustrate how regular exercise might alter features of T cell success contained within the cancer immunogram, which in turn may explain how regular exercise maintains immune control of immunogenic tumours. Each candidate component is driven by the effects of immunoregulatory cytokines (e.g., IL-7, IL-15), secreted from skeletal muscle in people who are physically active, on immune competency. The candidate components included in this model are: (1) Physical activity may alter general T cell status in blood by preserving naïve T cell frequency in ageing, thus enabling T cells to identify a greater diversity of tumour antigens in older age. As outlined in Determinant 1 however, we note that there is not conclusive evidence yet that naïve T cell output is maintained by physical activity in ageing. Instead, following antigen encounter, regular exercise may revert antigen-experienced memory T cells and preserve these as stem cell-like cells such as T_NRM_ cells and T_SCM_ cells, with a surface phenotype similar to naïve T cells. This maintenance of stem cell-like T cells (such as T_SCM_ and T_NRM_ cells) may augment the supplementation of tissue-associated memory T cells, thus leading to more persistent anti-tumour responses when required. (2) Acute exercise may augment the redistribution and infiltration of CD8^+^ T_EFF_ cells, as well as other T cell subsets, to tissue sites harbouring tumour antigens. However, as outlined in Determinant 2, we note that there are incompatibilities in the theory that exercise acutely mobilises effector cells to sites harbouring tumours. (3) Regular exercise may alter immune checkpoint expression, by preferentially suppressing development of CD4^+^ T_REG_ cells in tissue sites harbouring tumour antigens, averting T cell anergy and leading to more robust effector responses against cancer cells. In addition, regular exercise may reverse exhaustion to PD1^+^ stem cell-like CD8^+^ T cells – upon chronic exposure to antigen and immunosuppressive cytokines – which may help supplement the frequency of tissue-associated memory T cells with capacity to elicit persistent effector responses. (4) In tandem to Determinant 3, regular exercise may alter the presence of inflammatory mediators within the tumour microenvironment – namely IL-10 and TGF-β – by suppressing the development of T_REG_ *vs.* T_EFF_, thus alleviating immunosuppressive signalling within the tumour microenviornment to enhance T cell killing of cancer cells. (5) Exercise training may alter metabolic inhibitors of T cell activity within the tumour microenvironment, *via* the enhancements to immune competency discussed in Determinants 1-3, which increase T cell killing of cancer cells to reduce lactate accumulation, acidosis, and hypoxia which arise from the tumour cells themselves. Importantly, each component in this model is dependent on tumour-intrinsic factors: (A) immunogenicity (i.e., tumour ‘foreignness’), and (B) MHC-1 expression (i.e., ‘visibility’), and therefore the anti-cancer benefits of physical activity are unlikely to be seen in the absence of tumour immunogenicity and MHC-1 expression. T_REG_ cells, Regulatory T cells; T_EFF_ cells, Effector T cells; T_SCM_ cells, Stem-like T cells; T_NRM_ cells, Naïve-revertant memory T cells. Created with BioRender.com.

In Part 3, we appraised five Determinants which have each been used to independently explain how physical activity augments anti-cancer immunity. In doing so, it appears that these Determinants exhibit related features that are intertwined (**Figure 2**). A key overlapping feature of this appraisal is the apparent augmented regulation of T cells in the tumour microenvironment by physical activity.

In Determinants 1-3, it was highlighted that this process may be governed by immunoregulatory cytokines, such as skeletal muscle-derived IL-7 and IL-15. These immunoregulatory cytokines exert diverse immunoregulatory effects on T cells that change the fate of the cancer immunoediting process, which may lead to the exercise-induced maintenance of cancer in equilibrium, thus delaying and/or averting a clinical cancer diagnosis. Specifically, IL-7 and IL-15 in physically active people may regulate the phenotype and function of naïve T cells (102, 109, 127), which culminates in greater T cell diversity with ageing. Moreover, these so-called myokines may also preserve early differentiated memory T cells – such as T_SCM_ cells (148, 149) and T_NRM_ cells (152) – which possess features of plasticity, stemness and importantly, multipotent proliferative capacity and the propensity to migrate to tissues. Furthermore, IL-7 and IL-15 aid the development and maintenance of tissue-associated effector cells residing in close proximity to cancer development sites (185, 186) and exhausted T cells may regain function and eradicate cancer cells in response to IL-7 (229). Additionally, in the tissues, IL-15 preferentially supports the function and survival of effector T cells compared with T_REG_ cells (196) and blunts immunosuppressive functions of T_REG_ cells (197), thus reducing anergy. As a consequence of these immunophenotypic changes in the tumour – and as appraised in Determinant 4 – there is a shift in the cytokine milieu that corresponds with reductions to immunosuppressive cytokines including IL-10 and TGF-β, which favours effector competency. Additionally, as appraised in Determinant 5, and again as a consequence of immunophenotypic changes in the tumour and favourable effector competency, there is a shift in the metabolic profile away from accumulation of inhibitory factors including lactate, acidosis, glucose depletion, and hypoxia.

These observations indicate that T cell dysfunction in the tissues may be reversed by immunoregulatory cytokines in physically active individuals in a diverse yet interwoven manner, and future research is required to confirm that exercise – and, specifically, muscle – yields these beneficial effects. Whilst the five Determinants discussed in Part 3 demonstrate hypothetical synergy, the factor(s) ultimately responsible for augmenting T cell function against immunogenic cancer cells in physically active people remains unknown, and future research is needed to elucidate how this is ultimately achieved.

## Conclusion

Overall, there is consistent evidence that leading a physically active lifestyle reduces the risk of developing clinically diagnosed cancer across a multitude of different anatomical locations. A series of observations here indicate that regular exercise augments immune function – and specifically T cells – and this in turn should be considered an integral mechanism explaining reduced clinical cancer incidence in physically active people. Indeed, exercise-induced immuno-modulation likely explains why epidemiology evidence shows that physical activity does not prevent *de novo* neoplasia but instead reduces the incidence of more advanced disease (e.g., a clinical cancer diagnosis). Complementing these observations, it is apparent that the efficacy of physical activity appears to correspond with the tumour mutational (i.e., neoantigen) burden exhibited by different cancer types, which strongly implicates adaptive immunity – and specifically T cells – as a key anti-cancer mechanism of physical activity. We therefore hypothesise that physical activity augments T cell competency to promote the elimination of cancer cells after immunogenic mutational events have arisen, which in turn facilitates the maintenance of “covert” cancer within the equilibrium phase of the immunoediting process, thus delaying or averting a clinical cancer diagnosis. After synthesising different factors that determine T cell success against immunogenic cancer cells, we highlight that a possible unifying mechanism linking physical activity with improved T cell competency is the presence of elevated immunoregulatory cytokines – including the myokines IL-7 and IL-15 – in physically active people, which may be important in changing the trajectory of immunoediting by altering the fate of tissue-associated effector and regulatory T cells. However, whether T cell competency is augmented *via* the aforementioned mechanism, or *via* another exercise-induced factor, requires confirmation in future research.

## Author Contributions

AE, JC, JT and SM contributed to conception and design of the manuscript. AE wrote the first draft of the manuscript. JC wrote sections of the manuscript. All authors contributed to manuscript revision, read, and approved the submitted version.

## Funding

The University of Bath Open Access Fund funded open access publication fees.

## Conflict of Interest

The authors declare that the research was conducted in the absence of any commercial or financial relationships that could be construed as a potential conflict of interest.

## Publisher’s Note

All claims expressed in this article are solely those of the authors and do not necessarily represent those of their affiliated organizations, or those of the publisher, the editors and the reviewers. Any product that may be evaluated in this article, or claim that may be made by its manufacturer, is not guaranteed or endorsed by the publisher.

## Footnotes

1 Metabolic equivalent of task (MET) = a measure of energy expenditure required to perform a specific task. 1 MET = the energy expended when sitting quietly = 3.5 mL of oxygen/kg/min or approximately 1 Kcal/min. Energy expended when performing a specific activity for 1 hour divided by 3.5 = MET score. These scores are published in the Compendium of Physical Activities (15). MET-hours combine the MET score with the duration the activity is performed for. For example, brisk walking (4 METs) performed for 2 hours = 8 MET-hours of activity.

2 CV% calculated from published group means for pre- and post-intervention using the equation: ((mean of ‘group mean pre-intervention PSA’ and ‘group mean post-intervention PSA’) ÷ (SD between ‘group mean pre-intervention PSA’ and ‘group mean post-intervention PSA’)) × 100.
